# Alcohol and mortality in Mexico: prospective study of 150 000 adults

**DOI:** 10.1016/S2468-2667(24)00228-7

**Published:** 2024-11-01

**Authors:** Eirini Trichia, Jesus Alegre-Díaz, Diego Aguilar-Ramirez, Raúl Ramirez-Reyes, Adrián Garcilazo-Ávila, Carlos González-Carballo, Fiona Bragg, Louisa Gnatiuc Friedrichs, William G Herrington, Lisa Holland, Jason Torres, Rachel Wade, Rory Collins, Richard Peto, Jaime Berumen, Roberto Tapia-Conyer, Pablo Kuri-Morales, Jonathan R Emberson

**Affiliations:** Clinical Trial Service Unit & Epidemiological Studies Unit, Nuffield Department of Population Health, University of Oxford, Oxford, UK; Experimental Research Unit, National Autonomous University of Mexico, Mexico City, Mexico; Clinical Trial Service Unit & Epidemiological Studies Unit, Nuffield Department of Population Health, University of Oxford, Oxford, UK; Experimental Research Unit, National Autonomous University of Mexico, Mexico City, Mexico; Experimental Research Unit, National Autonomous University of Mexico, Mexico City, Mexico; Experimental Research Unit, National Autonomous University of Mexico, Mexico City, Mexico; Health Data Research UK Oxford, and Clinical Trial Service Unit & Epidemiological Studies Unit, Nuffield Department of Population Health, University of Oxford, Oxford, UK; Clinical Trial Service Unit & Epidemiological Studies Unit, Nuffield Department of Population Health, University of Oxford, Oxford, UK; Clinical Trial Service Unit & Epidemiological Studies Unit, Nuffield Department of Population Health, University of Oxford, Oxford, UK; Clinical Trial Service Unit & Epidemiological Studies Unit, Nuffield Department of Population Health, University of Oxford, Oxford, UK; Clinical Trial Service Unit & Epidemiological Studies Unit, Nuffield Department of Population Health, University of Oxford, Oxford, UK; Clinical Trial Service Unit & Epidemiological Studies Unit, Nuffield Department of Population Health, University of Oxford, Oxford, UK; Clinical Trial Service Unit & Epidemiological Studies Unit, Nuffield Department of Population Health, University of Oxford, Oxford, UK; Clinical Trial Service Unit & Epidemiological Studies Unit, Nuffield Department of Population Health, University of Oxford, Oxford, UK; Experimental Research Unit, National Autonomous University of Mexico, Mexico City, Mexico; Faculty of Medicine, National Autonomous University of Mexico, Mexico City, Mexico; Faculty of Medicine, National Autonomous University of Mexico, Mexico City, Mexico; Instituto Tecnológico y de Estudios Superiores de Monterrey, Monterrey, Mexico; Clinical Trial Service Unit & Epidemiological Studies Unit, Nuffield Department of Population Health, University of Oxford, Oxford, UK

## Abstract

**Background:**

Alcohol consumption is a leading cause of premature death globally, but there is no large-scale prospective evidence from Mexico.

**Methods:**

The Mexico City Prospective Study recruited 150 000 adults aged 35 years or older between 1998 and 2004. Participants were followed up until Oct 1, 2022 for cause-specific mortality. Cox regression in those with no self-reported chronic disease at entry (adjusted for age, sex, district, education, physical activity, smoking, and diabetes) was used to relate baseline-reported alcohol consumption (never, former, occasional [less than monthly], and regular [at least monthly, split into <70, ≥70 to <140, ≥140 to <210, and ≥210 g/week]) to mortality at ages 35−74 from all causes, and from a pre-specified alcohol-related set of underlying causes. Heavy episodic drinking (normally consuming >5 [men] or >4 [women] drinks on a single occasion) and type of preferred drink were also examined.

**Findings:**

Among 138 413 participants aged 35−74 years at recruitment, 21 136 (15%) were regular alcohol drinkers (14 863 [33%] men, 6273 [7%] women), of whom 13 383 (63%) favoured spirits and 6580 (31%) favoured beer. During follow-up, there were 13 889 deaths at ages 35−74 years, including 3067 deaths from the pre-specified alcohol-related causes. Overall, J-shaped associations with mortality were observed. Compared with occasional drinkers, those with baseline-reported consumption ≥210 g/week had 43% higher all-cause mortality (rate ratio [RR] 1·43 [95% CI 1·30−1·56]) and nearly three times the mortality from the pre-specified alcohol-related causes (2·77 [2·39−3·20]). Death from liver disease was strongly related to alcohol consumption; the RR comparing regular drinkers of ≥140 g/week with occasional drinkers was 4·03 (3·36−4·83). Compared with occasional light drinking, occasional heavy episodic drinking was associated with 20% higher alcohol-related mortality (1·20 [1·06−1·35]), and regular heavy episodic drinking was associated with 89% higher alcohol-related mortality (1·89 [1·67−2·15]). Drinks with alcohol percentages higher than spirits were associated with the greatest increased mortality risk, even after accounting for the total alcohol consumed.

**Interpretation:**

In this Mexican population, higher alcohol consumption, episodic drinking, and very high percentage alcoholic products were all associated with increased mortality.

**Funding:**

Wellcome Trust, the Mexican Health Ministry, the National Council of Science and Technology for Mexico, Cancer Research UK, British Heart Foundation, and the UK Medical Research Council.

## Introduction

Alcohol consumption is a leading preventable cause of death worldwide, estimated to account for 3 million deaths and over 130 million disability-adjusted life-years lost in 2016, with more than half of all alcohol-attributable deaths occurring in people younger than 60 years.^1^ Previous epidemiological studies in mostly high-income populations have provided evidence of the hazards of alcohol consumption for several diseases.^2^ Evidence triangulation, combining observational cohort and case control studies with genetic epidemiological approaches, has provided additional evidence on causality for several diseases,^3^ suggesting dose−response associations of higher alcohol consumption with higher risk of disease and mortality.

In Mexico, average per-capita yearly alcohol consumption among adults in 2017 was 10·3 L pure alcohol in men and 2·8 L pure alcohol in women,^4^ with 57% of Mexican men and 39% of Mexican women self-reporting as current alcohol drinkers.^5^ Although typical of many other countries in this regard, hazardous patterns of alcohol consumption are particularly common in Mexico. According to the Pan American Health Organization’s 2020 report on alcohol and health in the Americas, 31% of men and 6% of women older than 15 years regularly engaged in heavy episodic drinking.^6^ However, direct evidence from prospective studies in Mexico (and other Latin American populations) of the effect of alcohol consumption on mortality risk is scarce. In one study of 120 000 adults in Cuba, each additional 35 cL bottle of rum per week (110 g of pure alcohol) was associated with about 10% higher risk of all-cause mortality.^7^ Previous studies from Mexico include modelling studies derived from national surveys^8^ and reports of death rates for causes presumed to be alcohol-related.^9,10^ Estimates of the burden of disease attributed to alcohol consumption in Mexico still tend to rely on extrapolations from results derived in other populations,^11^ but such extrapolations might be unreliable for several reasons. Indeed, we have previously documented how the effect of both smoking^12^ and diabetes^13^ on mortality differs in Mexico compared with other populations.

Using data from the Mexico City Prospective Study, we aimed to assess the association of alcohol consumption with all-cause and alcohol-related mortality in 150 000 Mexican adults who have been followed up for approximately two decades.

## Methods

### Study design and participants

The Mexico City Prospective Study design, methods, and population have been described previously.^14^ Between 1998 and 2004, 52 644 men and 107 111 women residing in two contiguous districts in Mexico City (Coyoacán and Iztapalapa) were recruited at their homes. Using standardised procedures, trained nurses collected information on sociodemographic characteristics, lifestyle factors, and medical history. Physical measurements including blood pressure, weight, height, waist and hip circumference, and a blood sample were taken. Nuclear Magnetic Resonance metabolomics were measured in blood plasma using the Nightingale Health platform.^15^ To assess the extent to which exposures (including alcohol consumption) varied over time, a resurvey of 10 144 surviving participants took place in 2015−19. The resurvey involved repeat questionnaire data, physical measures, and the collection of blood and urine samples. Ethics approval was obtained from the Mexican Ministry of Health, the Mexican National Council of Science and Technology (reference 0595P-M), and the University of Oxford (reference C99.260). All participants provided written informed consent.

### Procedures

At baseline, participants self-reported their frequency of alcohol consumption over the past 12 months and, for those reporting any alcohol consumption, the number of glasses they would normally drink in a single occasion and their most frequently consumed type of drink. Types of drink included beer, wine, spirits (ie, brandy, whiskey, tequila, or rum), products with higher alcohol percentages (eg, traditional and local products such as aguardiente with up to 60% alcohol content, home distilled products with varying high alcohol content, or rectified spirits with up to 95% alcohol content), and other less frequently consumed drinks (eg, pulque or agave wine with up to 7% alcohol content, cooler, or other non-specified drinks). For those who reported to drink at least monthly, weekly alcohol consumption was estimated by multiplying the weekly number of glasses consumed by the average alcohol content for the beverage type they reported to drink most often. For this calculation, typical glass sizes and alcohol contents for Mexico were assumed (appendix 2 p 4).

Death registration in Mexico City is reliable and complete, with almost all deaths certified medically. Participants are followed up for cause-specific mortality through probabilistic linkage (based on name, including phonetic coding of names, age, and sex) to the Mexican System for Epidemiologic Death Statistics (Subsistema Epidemiológico y Estadístico de Defunciones [SEED]) electronic death registry in Mexico City, administered by the Ministry of Health. Field worker follow-up of more than 7000 deaths matched in this way confirmed that the match was correct in more than 95% of cases. Diseases recorded on death certificates were coded using the International Statistical Classification of Diseases and Related Health Problems, Tenth Revision, with subsequent review by study clinicians to recode, when necessary, the underlying cause of death.^13^ Participant deaths were tracked from recruitment (1998−2004) until Oct 1, 2022. The main analyses focused on all-cause mortality and a pre-specified alcohol-related set of underlying causes of death (appendix 2 p 5).^16^ Additional analyses were also performed for other causes of death (appendix 2 p 6).

### Statistical analysis

Analyses excluded participants aged 90 years or older and those with missing alcohol data, missing covariate data, or uncertain mortality linkage (defined as a ≥1 year discrepancy in a participant’s date of birth as recorded at the baseline survey compared with the matched death certificate). To limit the effects of reverse causation bias (whereby individuals with chronic disease reduce their alcohol consumption), the main analyses also excluded those with self-reported coronary heart disease, stroke, chronic kidney disease, cirrhosis, emphysema, or cancer at baseline.

For the main analyses, participants were classified into seven baseline-defined groups: never drinkers, former drinkers, occasional drinkers (defined as drinking less than monthly), and four groups of regular drinkers (monthly to less than weekly or weekly <70 g/week, ≥70 to <140 g/week, ≥140 to <210 g/week, and ≥210 g/week). 140 g pure alcohol is about equivalent to 3 L beer, 1·5 bottles of wine, or 350 mL spirits. Consistency in self-reported alcohol consumption between the baseline assessment and resurvey (capturing both changes in and stability of the baseline assessment) was assessed. Some analyses classified regular drinkers into fewer groups according to their weekly alcohol consumption (<140 g/week *vs* ≥140 g/week). Additional analyses investigated the relevance to death of frequency of drinking (never, former, 1−5 times per year, 6−11 times per year, 1 time per month, 2−3 times per month, 1−2 times per week, 3−4 times per week, and daily); heavy episodic drinking (defined for both occasional and regular drinkers as typically consuming >5 drinks for men or >4 drinks for women during a single occasion^17^); and type of alcoholic product consumed most frequently (wine, beer, spirits, products with higher alcohol percentages than spirits, and other). Analyses of type of alcohol were also performed separately among occasional and regular drinkers, with beer drinkers used as the reference category.

Cox proportional hazards regression models, with time since entry into the study as the underlying timescale, were used to assess the relevance of the baseline-reported alcohol consumption groups to all-cause and cause-specific mortality. The log hazard ratio from a Cox model provides a useful summary statistic for the average log mortality rate ratio (RR) across different time periods of follow-up. These mortality RRs were stratified by age-at-risk (5-year groups) and district of residence, and were adjusted for self-reported sex, educational attainment (university or college, high school, elementary school, or other), smoking (never, former, less than daily, >0 to <10 cigarettes per day, or ≥10 cigarettes per day), leisure time physical activity (none, up to 2 times per week, or ≥3 times per week), and self-reported diabetes at baseline. This approach allows for a different baseline hazard in each stratum, so the proportional hazards assumption was made only within strata of age-at-risk and residential district. For the main comparisons, the reference group was occasional drinkers; this was the largest single category in both men and women and one that was unlikely to be affected by reporting or mis-classification biases. For plotting, group-specific CIs around the RRs (including for the reference group with RR of 1·0) were estimated using the variance of the log risk in each group.^18^ Participants who did not die from the cause of interest were censored at the earliest of death from any other cause, the end of the age-at-risk period of interest, or Oct 1, 2022. The main analyses examined premature mortality (defined as death before age 75 years).

Additional analyses included analyses by age-at-risk (35−54 years and 55−74 years) and sex. Sensitivity analyses included exclusion of current or former smokers and participants with self-reported diabetes at recruitment, exclusion of the first 5 years of follow-up, inclusion of participants with self-reported chronic disease at baseline, inclusion of participants and deaths up to age 90 years, extension of the definition of alcohol-related causes to include breast and oesophageal cancers,^19^ adjustment for additional covariates (income; fruit and vegetable consumption; previously-diagnosed hypertension; and antihypertensive, antithrombotic, lipid-lowering, and respiratory medication use), and examination of RRs separately for the first 10 years of follow-up compared with later years.

See Online for appendix 2

Analyses were done in SAS version 9.4 and the figures were plotted in R version 4.2.2.

### Role of the funding source

The funders had no role in study design, data collection, analysis or interpretation, or writing of the report.

## Results

Of the 159 755 participants recruited, 11 862 (7%) were excluded from the present analyses. These comprised 854 (0·5%) aged 90 years or older at recruitment, a further 272 (0·2%) with missing alcohol or covariate data, a further 2629 (1·6%) with uncertain mortality linkage, a further 7892 (5%) with self-reported chronic diseases at baseline, and a further 215 (0·1%) who were recruited twice (data from the first visit at which a blood sample was collected were used for these participants). Of the remaining 147 893 participants, 138 413 (94%) were aged 35−74 years and 9480 (6%) were aged 75−89 years at recruitment.

Among participants aged 35−74 years at recruitment, 45 065 (33%) were men, 93 348 (67%) were women, and the mean age was 50·4 years (SD 10·8; table 1). 26 544 (19%) reported to be never drinkers, 17 629 (13%) were former drinkers, 73 104 (53%) were occasional drinkers, and 21 136 (15%) were regular drinkers.

Among the regular drinkers, median consumption was 48 g (IQR 24−133) of alcohol per week (6 alcoholic units or about 3 standard drinks per week). 12 188 (58% of regular drinkers) were in the <70 g per week category, 3677 (17%) were in the ≥70 to <140 g per week category, 2057 (10%) were in the ≥140 to <210 g per week category, and 3214 (15%) were in the ≥210 g per week category. In each category of current or former drinkers, the most commonly reported preferred alcoholic product was spirits (13 383 [63%] of regular drinkers), with beer being the second most popular (6580 [31%]; appendix 2 p 7).

**Figure 1:**
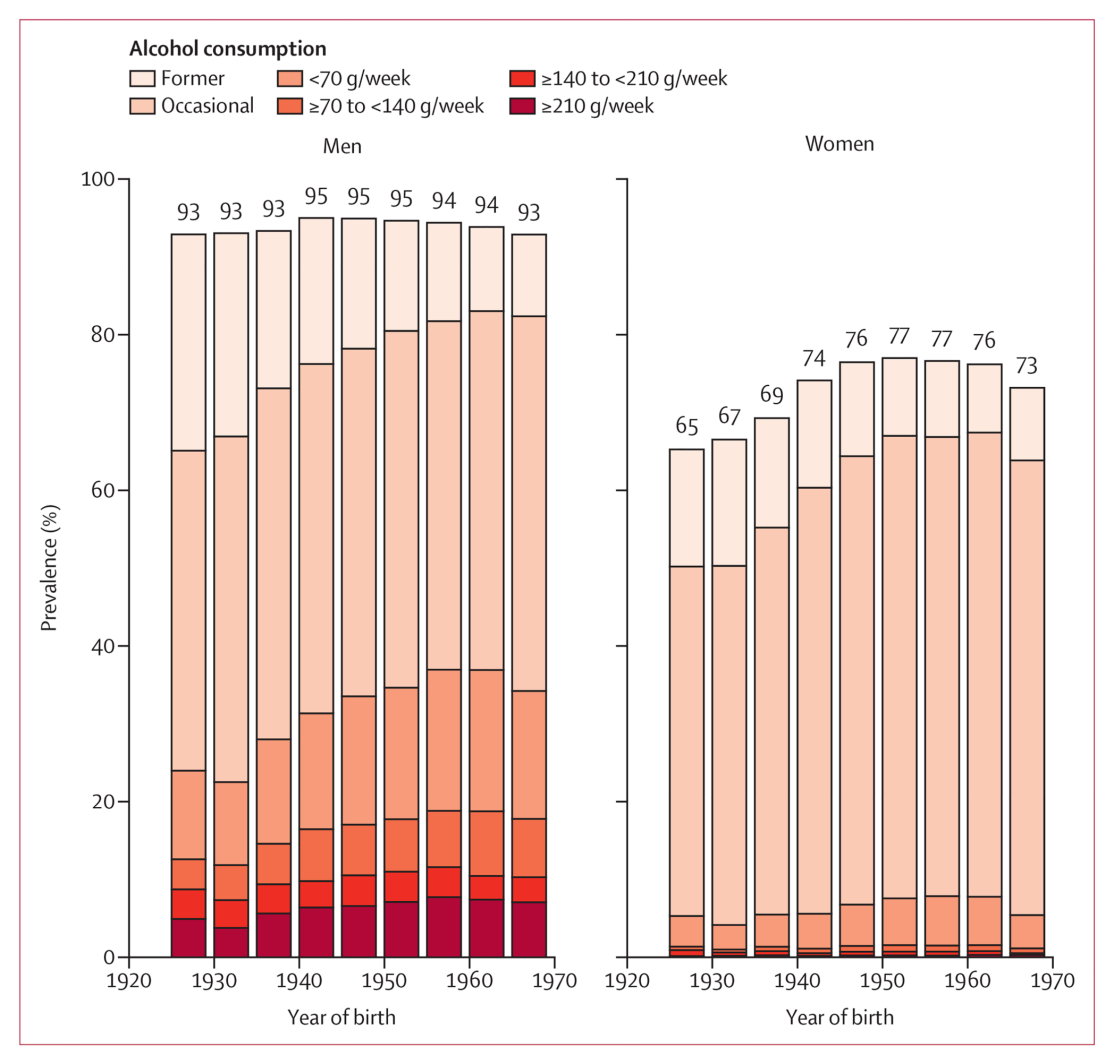
Alcohol consumption by year of birth Bars correspond to percentages of participants that ever drunk alcohol in nine separate birth cohorts (pre-1930 to 1965−69).

Regular drinkers were much more likely to be men than women (table 1, figure 1); 14 863 (33%) of the 45 065 men and 6273 (7%) of the 93 348 women were classified as regular drinkers. Regular drinkers were slightly younger than never drinkers, reflecting the moderate upward trend in alcohol consumption by year of birth (in both sexes, but especially in men). Median alcohol consumption in men who drank regularly was nearly treble that of women who drank regularly (appendix 2 pp 8−9). Regular drinkers were more likely to be residents of Coyoacán (the more affluent district), have higher income (among men), and to have attended university than never drinkers (table 1; appendix 2 p 10). In both men and women, regular drinkers were more likely to smoke cigarettes than non-drinkers; in women, regular drinkers were more likely to do leisure time physical activity than non-drinkers. Mean BMI was highest among the never drinkers, whereas mean waist-to-hip ratio was highest among those in the highest consumption category of regular drinkers. This finding was driven by the varying proportions of men across the alcohol consumption categories. Blood pressure increased modestly across the four regular drinking categories. Former and never drinkers were more likely to have diabetes and self-reported hypertension than occasional or regular drinkers. Blood lipids and medication use did not vary much between the groups.

When 9885 participants were resurveyed an average of 15 years later, one quarter of those who reported to be never drinkers at recruitment reported to be current drinkers at resurvey (almost all reporting to drink less than weekly; appendix 2 p 11). Of those who reported to be occasional drinkers at recruitment, very few (115 participants [2%]) reported higher amounts of consumption (ie, weekly drinking) at resurvey. However, of those who reported to be regular drinkers at baseline, less than one quarter remained current weekly drinkers at resurvey (with most reporting at resurvey to drink less than weekly). Consequently, median weekly alcohol consumption at resurvey for those who were regular drinkers at baseline and remained current drinkers at resurvey was lower. Even for the baseline-defined category with the highest reported alcohol consumption, median weekly alcohol consumption was just 48 g per week at resurvey (IQR 25−112).

**Figure 2:**
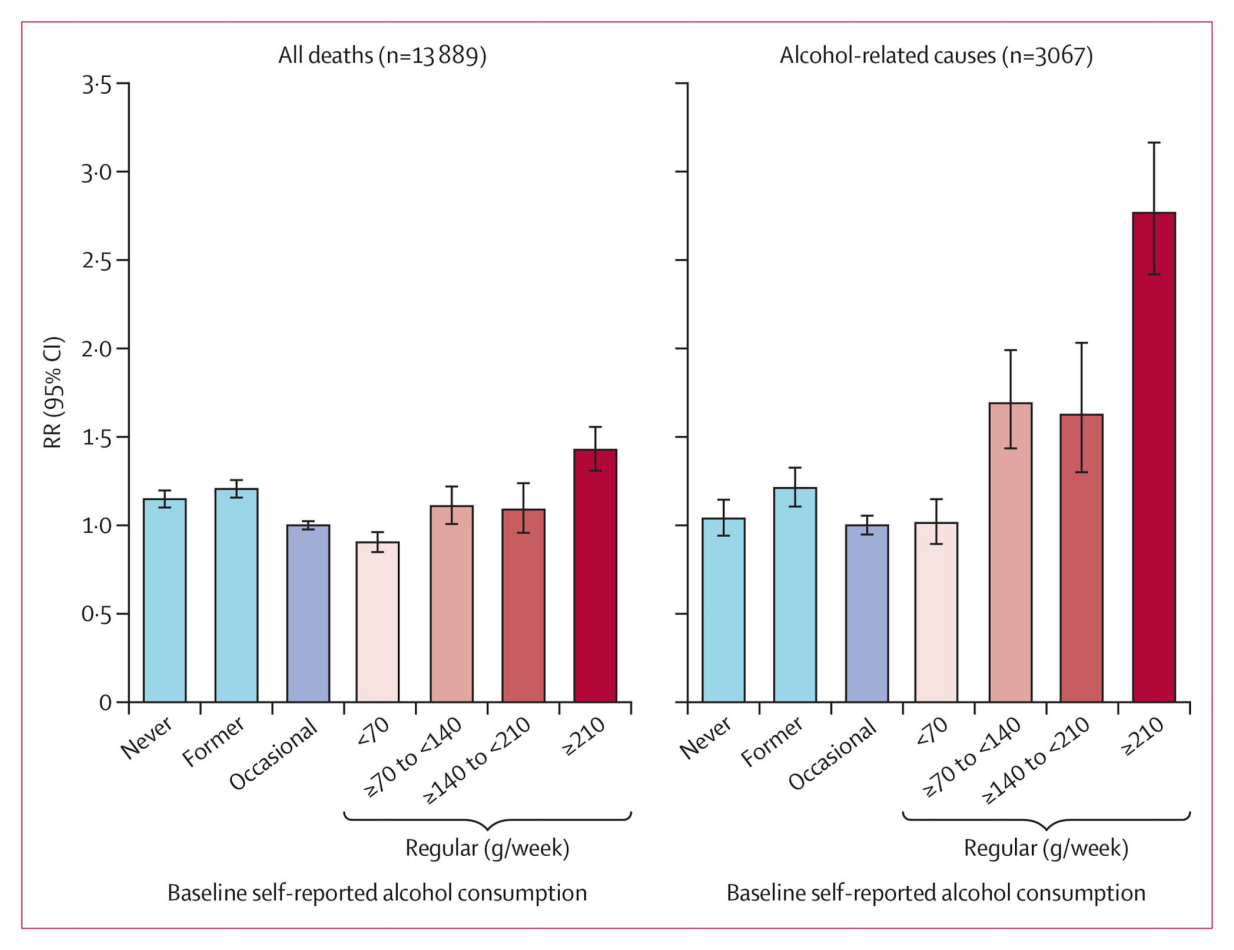
Alcohol consumption amount and risk of all-cause and alcohol-related mortality at ages 35−74 years Analyses are stratified by age-at-risk and district and adjusted for sex, education level, smoking, physical activity, and self-reported diabetes at baseline. Occasional drinkers are defined as those who reported drinking less than monthly. Regular drinkers are defined as those consuming alcohol at least on a monthly basis. The vertical lines through the top of each bar represent the group-specific 95% CIs. RR=mortality rate ratio.

During a median follow-up of 20·4 years (IQR 19·5−21·6), 13 889 participants died at ages 35−74 years. 3067 of these deaths were due to the prespecified group of alcohol-related causes (including 1161 liver deaths, 1018 respiratory deaths, 402 external deaths, and 486 deaths from other pre-specified causes; appendix 2 p 5). Overall, there were J-shaped associations between alcohol consumption and all-cause and alcoholrelated mortality (table 2, figure 2).

Among regular drinkers, all-cause and alcohol-related mortality increased with higher alcohol consumption. Compared with occasional drinkers, regular drinkers whose baseline-reported alcohol consumption was more than 210 g per week had a 43% higher all-cause mortality rate (RR 1·43 [95% CI 1·30−1·56]) and nearly three times the mortality rate from the diseases pre-specified as alcohol-related (2·77 [2·39−3·20]). For all-cause mortality, the adjusted death rate was lowest for regular drinkers who reported drinking <70 g per week. Mortality rates for never and former drinkers were higher than for occasional drinkers. Associations were broadly similar in men and women (appendix 2 p 12) and in all sensitivity analyses (appendix 2 pp 13−14). For both all-cause mortality and alcohol-related mortality, RRs were more extreme for deaths before age 55 years than for deaths at ages 55−74 years (appendix 2 p 15). Indeed, for deaths before age 55 years, regular drinkers of ≥140 g per week had over twice the mortality rate and over four times the death rate from alcohol-related diseases compared with occasional drinkers. Partly as a consequence, relative risks were also larger for deaths within the first 10 years of follow-up than for later years (appendix 2 p 16).

Consistent with the main analyses, higher self-reported frequencies of alcohol consumption were also associated with increased all-cause and alcohol-related mortality (appendix 2 p 3). For example, compared with those who reported to drink just 1−5 times a year, those who reported to drink daily had 74% higher all-cause mortality (RR 1·74 [95% CI 1·54−1·96]) and over four times the mortality rate from diseases pre-specified as likely to be alcohol-related (4·02 [3·36−4·81]). When subdividing the 94 240 occasional or regular drinkers by drinking pattern, compared with those who were classified as occasional light drinkers, those who were classified as regular heavy episodic drinkers had 20% higher all-cause mortality (1·20 [1·12−1·28]) and 89% higher mortality from the group of alcohol-related diseases (1·89 [1·67−2·15]; table 2). Among regular drinkers, those who reported to prefer higher alcohol percentage products to any other type of product had the highest all-cause and alcoholrelated mortality rates (table 2; appendix 2 p 17). This finding was explained partly but not wholly by differences in the estimated amount of weekly alcohol consumed between the groups. Among occasional drinkers there was little detectable variation in all-cause or alcohol-related mortality rates depending on preferred product, although occasional spirit drinkers seemed to have marginally lower mortality rates than occasional beer drinkers (appendix 2 p 17).

Of the diseases pre-specified to be most likely related to alcohol, liver disease was most strongly related, particularly for deaths at younger ages. The RR for death from liver disease comparing regular drinkers of ≥140 g per week with occasional drinkers was 4·03 (95% CI 3·36−4·83), whereas it was 8·07 (5·63−11·56) for such deaths before age 55 years (appendix 2 p 18). Higher amounts of alcohol consumption were also associated with higher risks of death from the pre-specified external and other causes of death, but not with higher risks of the pre-specified respiratory causes of death. For the causes of death that were not pre-specified to be most likely related to alcohol consumption, associations with alcohol consumption were modest in magnitude and variable in shape and direction (appendix 2 p 19). In particular, for vascular mortality, both regular drinkers of <140 g per week and ≥140 g per week had marginally lower risk than occasional drinkers, and substantially lower risk than never or former drinkers.

## Discussion

In this population of Mexican adults, for whom spirits and beer were the favoured drinks, higher alcohol consumption was related to higher mortality. Compared with occasional drinkers, the risk of death from any cause was increased by nearly one half for those who reported at baseline to drink more than the equivalent of about 500 mL of spirits per week. Consumption of products with alcohol percentages higher than 40%, although rare, was associated with the greatest excess mortality of the various beverage types.

Alcohol has been found to be associated with increased mortality in many previous studies. An individualparticipant data meta-analysis of 83 prospective cohort studies from 19 high-income countries identified that the lowest risk of mortality was at about 100 g of alcohol per week, above which there was a strong positive dose− response association of higher consumption with increased mortality.^20^ In Russia, case−control^21^ and prospective studies^16^ have found alcohol to be associated with substantial mortality increases, with hazardous alcohol drinking patterns potentially responsible for half of all deaths in working-age men in a typical Russian city.^21^ Evidence from studies in Latin America is scarce but, consistent with the findings in the present report, one large prospective study of Cuban adults found that weekly alcohol consumption was continuously related to premature mortality.^7^ In Mexico, a report that used open-access data from the Mexico City Prospective Study assessed the role of reverse causality bias when examining associations of various lifestyle factors with mortality risk; however, that report included only a limited range and depth of analyses of alcohol consumption.^22^ Of the individual causes of death, cardiovascular disease has generally been found to have a J-shaped association with alcohol consumption, reflecting an inverse association with coronary death and positive associations with stroke, heart failure, and other vascular causes of death.^20^ Strong positive associations of alcohol consumption with stroke have also been observed in China, along with increased risks of many other diseases.^3^ However, mendelian randomisation studies have indicated that genetic variants associated with non-drinking, lower alcohol consumption, and a lower prevalence of episodic drinking are associated with a lower risk of both coronary heart disease^23^ and stroke.^24^ The J-shaped associations between alcohol consumption and mortality risk in some observational studies might therefore reflect reverse causality bias (eg, former drinkers stopping or reducing because of ill-health), misclassification between never and former drinkers, or other potential biases.^25^ Heavy episodic drinking is common in Mexico,^6^ and our finding of increased mortality risk associated with occasional and, particularly, regular episodic drinking is consistent with previous studies.^26^

The underlying pathways linking alcohol consumption to the causes of death pre-specified to be likely to be alcohol-related include both biological and behavioural. By far the largest excess risks seen in the current analysis were for hepatobiliary disease, for which mechanisms are well described and include hepatic steatosis and fibrosis.^27^ For other alcohol-related causes (eg, external causes), behavioural changes due to alcohol consumption might be more relevant. Alcohol increases adiposity (both overall and in the liver), triglycerides, insulin resistance, and blood pressure.^28^ Alcohol also increases high-density lipoprotein cholesterol, although randomised trials^29^ and mendelian randomisation studies^30^ suggest that such increases would not lead to a reduction in cardioembolic diseases.

The key strength of the current study is the availability of both a large sample size and prolonged follow-up in a previously understudied population. We adjusted for available confounders, excluded participants with pre-existing disease (other than diabetes), and performed a range of sensitivity analyses (all of which gave results broadly consistent with the main analyses). Nevertheless, we cannot rule out some residual confounding due to important unmeasured confounders such as dietary habits, chronic stress, or other substance use, or due to imperfect assessment of measured confounders. We also cannot rule out some residual reverse causality bias due to misreporting of disease history. Never drinkers might have distinct characteristics and confounding patterns from the other groups that might not be fully captured, which could explain the higher mortality risk in that group. Alcohol consumption was self-reported, and hence prone to recall and social desirability bias. Although we deliberately used occasional drinkers as the reference category rather than never or former drinkers, some misclassification between these categories remains possible. Participants were classified based on a single assessment of alcohol consumption at study entry, and in a subset of participants resurveyed approximately 15 years later, alcohol consumption in each baseline-defined group was much lower than the baseline-reported amount. Consequently, regression dilution means that the increased mortality risks we observed with higher alcohol consumption categories probably relate to a much narrower range in so-called usual alcohol consumption than the range across the baseline-defined groups. Despite these limitations, we observed strong associations between the baseline-defined groups and alcohol-related causes (and especially death due to hepatobiliary disease). The study population arises from just two districts of Mexico City and so is not representative of adults throughout Mexico (or even Mexico City). Participants who agreed to take part might also have differed systematically from those who did not (including by their alcohol consumption). Additionally, data on ethnicity were not collected, so we could not assess representativeness from this perspective or investigate association by ethnic group. However, prospective studies of non-representative cohorts of individuals can still provide reliable evidence about the associations of risk factors with disease that are widely generalisable.^31^ Finally, an absence of information on non-fatal outcomes means that the conclusions apply directly only to causes of death.

The 2023 Guideline for a Healthy and Sustainable Diet in the Mexican Population recommends avoiding alcohol consumption.^32^ The results of the present study offer context for this recommendation by providing direct population-specific evidence on the hazards to health of alcohol consumption in Mexican adults. Policy interventions to reduce the average population amount of alcohol consumption as well as harmful patterns of alcohol consumption include taxation, increasing sobriety checkpoints to counter drink-driving, restricting the availability of alcohol to individuals at high risk, strengthening pricing policies targeting cheap alcohol to protect heavy drinkers and young people, and regulation of advertising.^33^

In summary, in this large prospective study of Mexican adults, larger quantity, more frequent, and episodic drinking, and drinking very high percentage alcoholic products, were associated with increased premature mortality. Public health policies aimed at reducing population alcohol consumption and harmful patterns of alcohol consumption could help reduce premature deaths in Mexican adults.

## Supplementary Material

Supplementary appendix

## Figures and Tables

**Table 1:** Baseline characteristics of 138 413 adults aged 35–74 years by self-reported alcohol consumption

	Never drinker (n=26 544)	Former drinker (n=17 629)	Occasional drinker (n=73 104)	Regular drinkers (g/week)				
				<70 (n=12 188)	≥70 to <140 (n=3677)	≥140 to <210 (n=2057)	≥210 (n=3214)	All regular (n=21 136)
Weekly amount of alcohol (g)	NA	NA	NA	28 (13-37)	84 (84-96)	144 (144-149)	288 (235-347)	48 (24-133)*
Preferred alcohol product								
Beer	NA	3440 (20%)	14 451 (20%)	3547 (29%)	1221 (33%)	723 (35%)	1089 (34%)	6580 (31%)
Wine	NA	662 (4%)	6219 (9%)	293 (2%)	46 (1%)	77 (4%)	15 (0%)	431 (2%)
Spirits	NA	12 039 (68%)	47 915 (66%)	8096 (66%)	2275(62%)	1219 (59%)	1793 (56%)	13383(63%)
Higher alcohol percentage products†	NA	208 (1%)	111 (0%)	23 (0%)	29 (1%)	5 (0%)	92 (3%)	149 (1%)
Other (eg, cooler or pulque)	NA	1049 (6%)	4347 (6%)	229 (2%)	106 (3%)	33 (2%)	225 (7%)	593 (3%)
Age, years	51·3 (11·4)	53·2 (11·2)	49·8 (10·4)	48·9 (10·0)	48·8 (10·1)	51·0 (10·7)	49·0 (10·1)	49·1 (10·1)
Sex								
Women	23 894 (90%)	10 467 (59%)	52 714 (72%)	4957(41%)	651 (18%)	440 (21%)	225 (7%)	6273 (30%)
Men	2650 (10%)	7162(41%)	20 390 (28%)	7231 (59%)	3026 (82%)	1617 (79%)	2989 (93%)	14 863 (70%)
Resident of Coyoacán	8392 (32%)	6652 (38%)	28 946 (40%)	6777 (56%)	1962 (53%)	1137 (55%)	1593 (50%)	11 469 (54%)
University or college educated	2973 (11%)	1851 (10%)	12 002 (16%)	3833 (31%)	1000 (27%)	610 (30%)	565 (18%)	6008 (28%)
Smoking behaviour								
Never smoker	20 273 (76%)	7074 (40%)	34 983 (48%)	3333 (27%)	589 (16%)	388 (19%)	429 (13%)	4739 (22%)
Ex-smoker	2859 (11%)	4944 (28%)	13 440 (18%)	2771 (23%)	821 (22%)	456 (22%)	617 (19%)	4665 (22%)
Current smoker	3412 (13%)	5611 (32%)	24 681 (34%)	6084 (50%)	2267(62%)	1213 (59%)	2168(67%)	11 732 (56%)
Regular leisure time physical activity‡	4417 (17%)	3526 (20%)	16 449 (23%)	4006 (33%)	1155 (31%)	677 (33%)	840 (26%)	6678 (32%)
Previously diagnosed diabetes	3961 (15%)	3391 (19%)	8352 (11%)	1018 (8%)	328 (9%)	189 (9%)	341 (11%)	1876 (9%)
Physical measurements								
BMI, kg/m^2^	29·5 (5·3)	29·2 (5·1)	29·3 (5·1)	28·1 (4·5)	28·1 (4·4)	27·7 (4·3)	27·8 (4·7)	28·0 (4·5)
Waist-hip ratio	0·89 (0·7)	0·91 (0·08)	0·89 (0·08)	0·91 (0·08)	0·93 (0·07)	0· 93 (0·07)	0·95 (0·07)	0·92 (0·08)
Systolic blood pressure, mm Hg	127·7 (17·2)	128·5 (17·1)	125·9 (15·8)	125·5 (15·0)	126·7 (14·7)	128·0 (15·7)	128·7 (15·4)	126·4 (15·1)
Diastolic blood pressure, mm Hg	83·0 (10·3)	83·6 (10·2)	82·7 (10·0)	82·9 (9·8)	83·9 (9·7)	84·3 (10·1)	85·0 (10·0)	83·5 (9·9)
Laboratory measurements§								
LDL cholesterol, mmol/L	2·44 (0·79)	2·41 (0·80)	2·47 (0·78)	2·50 (0·79)	2·46 (0·79)	2·46 (0·81)	2·35 (0·83)	2·47 (0·80)
HDL cholesterol, mmol/L	1·02 (0·21)	0·98 (0·21)	1·00 (0·21)	0·99 (0·21)	0·99 (0·22)	1·01 (0·23)	1·01 (0·25)	1·00 (0·22)
Triglycerides, mmol/L	1·55 (0·65)	1·58 (0·66)	1·57 (0·65)	1·59 (0·66)	1·68 (0·71)	1·71 (0·73)	1·77 (0·80)	1·64 (0·70)
Apolipoprotein A1, g/L	1·23 (0·19)	1·20 (0·19)	1·22 (0·19)	1·22 (0·19)	1·22 (0·20)	1·25 (0·21)	1·25 (0·23)	1·23 (0·20)
Apolipoprotein B, g/L	0·89 (0·21)	0·89 (0·22)	0·90 (0·21)	0·90 (0·21)	0·90 (0·21)	0·90 (0·21)	0·88 (0·22)	0·90 (0·21)

Results shown are n (%), mean (SD), or median (IQR). Occasional drinkers are those who reported drinking alcohol on a less than monthly basis. Regular drinkers are those who reported drinking at least monthly. Those who reported drinking monthly but less than weekly are included in the <70 g/week category. For reference, 140 g pure alcohol is about equivalent to 3 L beer, 1·5 bottles of wine, or 350 mL spirits. NA=not applicable.

* Among 14 863 regular drinkers (men), the median consumption was 76 (IQR 29–149) g/week, whereas among 6273 regular drinkers (women), the median consumption was 28 (12–48) g/week (appendix 2 pp 8−9).

† Listed as pure alcohol in the baseline questionnaire (assumed to correspond to products with alcohol percentages higher than spirits).

‡ At least 1 day/week. §Measured using the Nightingale Health nuclear magnetic resonance platform.

**Table 2:** Alcohol consumption amount, pattern, and preferred alcohol product and risk of all-cause and alcohol-related mortality at ages 35−74 years

	Participants, n	All deaths			Alcohol-related causes	
		Deaths, n	RR (95% CI)		Deaths, n	RR (95% CI)
**Alcohol consumption amount**						
Never	26 544	2597	1·15 (1·10−1·20)		477	1·04 (0·93−1·16)
Former	17 629	2373	1·21 (1·15−1·26)		479	1·21 (1·09−1·35)
Occasional	73 104	6656	1·00		1384	1·00
Regular (g/week)						
<70	12 188	1043	0·90 (0·85−0·97)		259	1·01 (0·88−1·16)
≥70 to <140	3677	438	1·11 (1·00−1·22)		151	1·69 (1·42−2·01)
≥140 to <210	2057	237	1·09 (0·96−1·24)		79	1·63 (1·29−2·05)
≥210	3214	545	1·43 (1·30−1·56)		238	2·77 (2·39−3·20)
**Alcohol drinking pattern, among drinkers**						
Occasional, light	57 785	4899	1·00		974	1·00
Occasional, heavy episodic*	15 319	1757	1·08 (1·02−1·14)		410	1·20 (1·06−1·35)
Regular, light	11 312	966	0·95 (0·89−1·02)		280	1·30 (1·13−1·49)
Regular, heavy episodic*	9824	1297	1·20 (1·12−1·28)		447	1·89 (1·67−2·15)
**Preferred alcohol product, among drinkers**						
Occasional	73 104	6656	1·00		1384	1·00
Regular						
Wine	431	30	0·89 (0·62−1·27)		15	2·07 (1·24−3·45)
Beer	6580	715	1·06 (0·98−1·14)		235	1·51 (1·31−1·74)
Spirits	13 383	1382	1·01 (0·95−1·07)		412	1·34 (1·20−1·51)
Higher alcohol percentage productst	149	38	2·31 (1·68−3·18)		27	6·80 (4·63−9·99)
Other (eg, cooler or pulque)	593	98	1·48 (1·21−1·81)		38	2·64 (1·91−3·65)
**Preferred alcohol product, adjusted for overall alcohol amount**						
Occasional	73 104	6656	1·00		1384	1·00
Regular						
Wine	431	30	0·82 (0·57−1·18)		15	1·78 (1·07−2·96)
Beer	6580	715	0·95 (0·87−1·03)		235	1·23 (1·06−1·43)
Spirits	13 383	1382	0·91 (0·86−0·97)		412	1·10 (0·98−1·25)
Higher alcohol percentage productst	149	38	1·49 (1·06−2·08)		27	2·74 (1·79−4·18)
Other (eg, cooler or pulque)	593	98	1·10 (0·89−1·36)		38	1·34 (0·93−1·93)

Occasional drinkers are those who reported drinking alcohol on a less than monthly basis. Regular drinkers are those who reported drinking at least monthly. Those who reported drinking monthly but less than weekly are included in the <70 g/week category. For reference, 140 g pure alcohol is about equivalent to 3 L beer, 1·5 bottles of wine, or 350 mL spirits.

* Heavy episodic drinking defined as >5 drinks (men) or >4 drinks (women) typically consumed per drinking session.

† Listed as pure alcohol in the baseline questionnaire (assumed to correspond to products with alcohol percentages higher than spirits). RR=mortality rate ratio.

## Data Availability

Data from the Mexico City Prospective Study are available to bona fide researchers. For more details, the study’s Data and Sample Sharing policy may be downloaded (in English or Spanish) from https://www.ctsu.ox.ac.uk/research/mcps. Available study data can be examined in detail through the study’s Data Showcase, available at https://datashare.ndph.ox.ac.uk/mexico/.

## Research in context

### Evidence before this study

We performed a literature search in PubMed to identify articles published from database inception to May 21, 2024 that reported on studies investigating associations of alcohol with mortality in Mexico or Latin America using the terms (“alcohol”) AND (“mortality” OR “death”) AND (“Mexico” OR “Mexican” OR “Latin America” OR “Latin American”). One report using openaccess data from the Mexico City Prospective Study assessed the role of reverse causality bias when examining associations of various lifestyle factors with mortality risk, but it included only a limited range and depth of analyses of alcohol consumption. Other studies from Mexico included modelling studies derived from national surveys or reports of death rates for causes presumed to be alcohol-related. A study of approximately 120 000 adults in Cuba reported a 10% increase in mortality risk for each additional bottle of rum per week, but the generalisability of such findings for Mexico (in which consumption, type, and pattern of drinking can differ) is unclear.

### Added value of this study

In the present study of approximately 150 000 individuals from Mexico City, with 13 889 deaths from any cause at ages 35−74 years, all-cause mortality increased across the four studied categories of regular alcohol consumption. Compared with occasional drinkers, those reporting at recruitment to drink ≥210 g of alcohol per week (equivalent to about 500 mL of spirits) had a 43% higher all-cause mortality rate and nearly three times the mortality rate from a subset of causes pre-specified as likely to be alcohol related. Liver disease death was especially strongly related to alcohol consumption; regular drinkers of ≥140 g/week had more than four times the mortality rate from liver disease than occasional drinkers. Compared with occasional light drinking, occasional heavy episodic drinking was associated with 20% higher alcohol-related mortality, and regular heavy episodic drinking was associated with 89% higher alcohol-related mortality. Consumption of products with alcohol percentages higher than spirits, although rare, was associated with the greatest mortality risk.

### Implications of all the available evidence

Higher alcohol consumption, episodic drinking, and very high percentage alcoholic products are associated with increased premature mortality in Mexican adults, particularly above the equivalent of more than half a litre of spirits per week. Policies addressing such behaviours could lead to a reduction in premature adult mortality in Mexico.
