# Supplementary material for: Alcohol and mortality in Mexico: prospective study of 150 000 adults
Source: Lancet Public Health. Author manuscript; Available in PMC 2024 Nov 21. (PMC7616839; doi:10.1016/S2468-2667(24)00228-7)
Supplement: Supplementary appendix [file EMS199879-supplement-Supplementary_appendix.pdf]

THE LANCET
Public Health

Supplementary appendix 1

This translation in Spanish was submitted by the authors and we reproduce it as supplied. It has not been peer reviewed. *The Lancet*'s editorial processes have only been applied to the original in English, which should serve as reference for this manuscript.

Los autores nos proporcionaron esta traducción al español y la reproducimos tal como nos fue entregada. No la hemos revisado. Los procesos editoriales de *The Lancet* se han aplicado únicamente al original en inglés, que debe servir de referencia para este manuscrito.

Supplement to: Trichia E, Alegre-Díaz J, Aguilar-Ramirez D, et al. Alcohol and mortality in Mexico: prospective study of 150 000 adults. *Lancet Public Health* 2024; **9:** e907–15.

Resumen

Antecedentes: El consumo de alcohol es una de las principales causas de muerte prematura a nivel mundial, pero no existe evidencia prospectiva a gran escala en México.

Metodología: El Estudio Prospectivo de la Ciudad de México reclutó a 150,000 adultos de 35 años o más entre 1998 y 2004. Los participantes fueron seguidos hasta el 1 de octubre de 2022 para determinar la mortalidad por causas específicas. Se utilizaron modelos de Cox en aquellos sin enfermedad crónica autoreportada (ajustados por edad, sexo, distrito de residencia, educación, actividad física, tabaquismo y diabetes) para relacionar el consumo de alcohol reportado al inicio (nunca, exbebedor, bebedor ocasional [menos de una vez al mes] y bebedor regular [al menos una vez al mes, dividido en <70, ≥70 a <140, ≥140 a <210 y ≥210 g/semana]) con la mortalidad a edades entre 35 y 74 años por todas las causas y por un conjunto preespecificado de causas subyacentes relacionadas con el alcohol. También se examinó el consumo episódico excesivo de alcohol (consumo habitual de más de 5 [hombres] o más de 4 [mujeres] bebidas en una sola ocasión) y el tipo de bebida preferida.

Resultados: De los 138,413 participantes cuya edad al inicio del estudio era entre 35 y 74 años, 21,136 (15%) eran bebedores regulares de alcohol (14,863 [33%] hombres, 6,273 [7%] mujeres), de los cuales 13,383 (63%) preferían destilados y 6,580 (31%) preferían cerveza. Durante el seguimiento, hubo 13,889 muertes a edades entre 35 a 74 años, incluyendo 3,067 muertes por las causas preespecificadas como relacionadas con el alcohol. En general, se observaron asociaciones en forma de J con la mortalidad. En comparación con los bebedores ocasionales, aquellos con consumo reportado al inicio del estudio de 210 g/semana o más tuvieron 43% más riesgo de mortalidad por todas las causas (razón de tasas [RR] 1.43 [IC del 95%: 1.30–1.56]) y casi tres veces más riesgo de mortalidad por las causas preespecificadas como relacionadas con el alcohol (2.77 [2.39–3.20]). La muerte por enfermedad hepática estuvo fuertemente relacionada con el consumo de alcohol; la RR comparando bebedores regulares de 140 g/semana o más con bebedores ocasionales fue de 4.03 (3.36–4.83). En comparación con el consumo ligero ocasional, el consumo episódico excesivo ocasional se asoció con 20% más riesgo de mortalidad relacionada con el alcohol (1.20 [1.06–1.35]), y el consumo episódico excesivo regular se asoció con 89% más riesgo de mortalidad relacionada con el alcohol (1.89 [1.67–2.15]). Las bebidas con porcentajes de alcohol más altos que los destilados se asociaron con el mayor aumento en el riesgo de mortalidad, incluso después de considerar el total de alcohol consumido.

Interpretación: En esta población mexicana, un mayor consumo de alcohol, el consumo episódico y productos alcohólicos con un porcentaje muy alto de alcohol se asociaron con un aumento en la mortalidad.

Financiamiento: Wellcome Trust, la Secretaría de Salud de México, el Consejo Nacional de Ciencia y Tecnología de México, Cancer Research UK, British Heart Foundation y UK Medical Research Council.

THE LANCET
Public Health

Supplementary appendix 2

This appendix formed part of the original submission and has been peer reviewed. We post it as supplied by the authors.

Supplement to: Trichia E, Alegre-Díaz J, Aguilar-Ramirez D, et al. Alcohol and mortality in Mexico: prospective study of 150 000 adults. *Lancet Public Health* 2024; **9:** e907–15.

Alcohol and mortality in Mexico: prospective study of 150 000 adults

Online appendix

Webfigures		Page
1.	All-cause and alcohol-related mortality risk at ages 35 to 74 years by frequency of alcohol consumption	3

Webtables		
1.	Assumed alcohol contents and volumes of a ‘standard drink’ for different types of alcohol products	4
2a.	ICD-10 codes of the causes of death prespecified as potentially alcohol-related	5
2b.	ICD-10 codes of the causes of death not prespecified as potentially alcohol-related	6
3.	Self-reported baseline alcohol consumption among 21 136 'regular' drinkers	7
4a.	Baseline characteristics of 45 065 men aged 35-74 years by self-reported alcohol consumption	8
4b.	Baseline characteristics of 93 348 women aged 35-74 years by self-reported alcohol consumption	9
5.	Additional baseline characteristics of participants aged 35-74 years by self-reported alcohol consumption	10
6.	Self-reported alcohol consumption at baseline and at resurvey ~15 years later	11
7.	Alcohol consumption versus all-cause and alcohol-related mortality at ages 35-74 years, by sex	12
8.	Sensitivity analyses	13
9.	Alcohol consumption versus all-cause and alcohol-related mortality at ages 35-74 years, overall and by age-at-risk	15
10.	Alcohol consumption versus all-cause and alcohol-related mortality at ages 35-74 years, by period of follow-up	16
11.	Alcohol product versus all-cause and alcohol-related mortality at ages 35-74 years in occasional and in regular drinkers, before and after adjustment for total alcohol consumed	17
12.	Alcohol consumption versus alcohol-related causes of death at ages 35-74 years, overall and by age-at-risk	18
13.	Alcohol consumption versus non-alcohol-related mortality at ages 35-74 years, overall and by age-at-risk	19

Webfigure 1. All−cause and alcohol−related mortality risk at ages 35 to 74 years by FREQUENCY of alcohol consumption

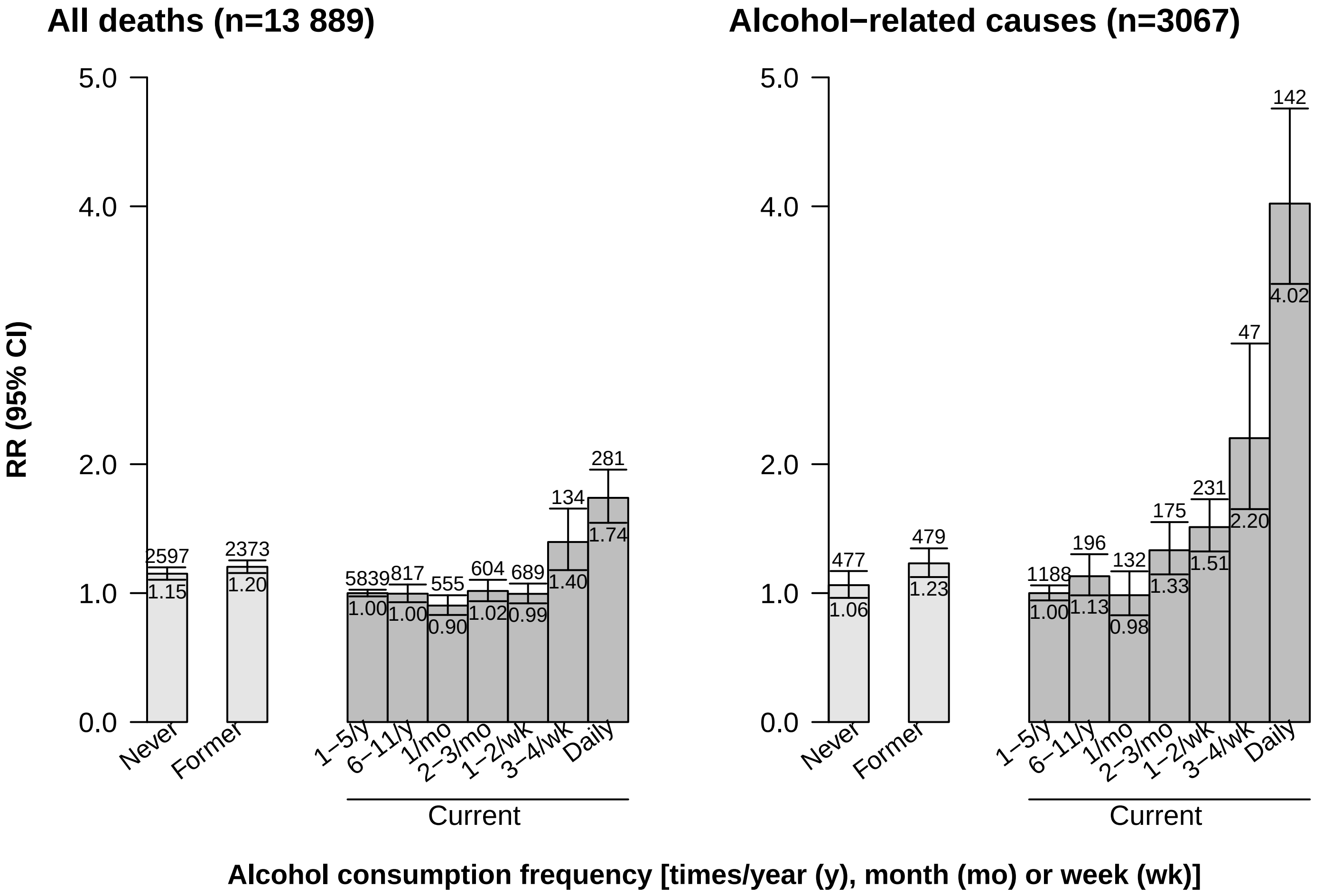

Adjustments and notation as for Figure 2. Compared with occasional drinkers (1−5 times/year), the all−cause mortality RR was 1.15 (1.10−1.21) for never drinkers, 1.20 (1.15−1.26) for former drinkers, 1.00 (0.92−1.07) for drinkers 6−11 times/year, 0.90 (0.83−0.99) for drinkers 1 time/month, 1.02 (0.93−1.11) for drinkers 2−3 times/month, 0.99 (0.92−1.08) for drinkers 1−2 times/week, 1.40 (1.18−1.66) for drinkers 3−4 times/week, and 1.74 (1.54−1.96) for daily drinkers. For deaths pre−specified as alcohol related these RRs were 1.06 (0.95−1.18), 1.23 (1.10−1.37), 1.13 (0.97−1.32), 0.98 (0.82−1.18), 1.33 (1.13−1.57), 1.51 (1.30−1.75), 2.20 (1.64−2.96), and 4.02 (3.36−4.81), respectively.

Webtable 1. Assumed alcohol contents and volumes of a 'standard drink' for different types of alcohol products

Alcohol product	Volume of standard drink (ml)*	Alcohol content, %
Beer	330	5
Wine	140	12
Spirits	40	40
Pulque	240	6
Cooler	340	5
Higher alcohol percentage products †	25	60
Other ‡	188	14

* Based on a 2017 report by the Social Investigations Foundation (*The Standard Drink in Mexico. A tool for the prevention of harmful use of alcohol*. https://www.tragoestandar.org.mx/wp-content/uploads/2020/11/The_Standard_Drink_In_Mexico.pdf [accessed 29th August 2024]).

† Listed as 'pure alcohol' in the baseline questionnaire, it is assumed that it refers to alcohol products with alcohol percentages higher than spirits.

‡ Alcohol content assumed to be the average across all products except the 'higher alcohol percentage products' category.

Webtable 2a. ICD-10 codes of the causes of death pre-specified* as potentially alcohol-related

Underlying cause	ICD-10 codes (number of deaths at ages 35-74 years)*
Alcohol poisoning (*n*=9)	F102 (7), X459 (1), Y159 (1)
Suicide (*n*=19)	X640 (1), X650 (1), X680 (1), X700 (6), X702 (1), X708 (1), X740 (3), X780 (3), X800 (1), X840 (1)
Assault (*n*=49)	X910 (1), X912 (1), X914 (2), X950 (3), X954 (19), X955 (1), X959 (1), X990 (8), X994 (5), X999 (2), Y044 (2), Y048 (1), Y084 (1), Y094 (1), Y099 (1)
Transport accidents (*n*=125)	V011 (1), V029 (1), V041 (1), V049 (1), V051 (1), V059 (1), V093 (3), V099 (72), V182 (1), V209 (1), V299 (1), V439 (1), V489 (1), V494 (1), V496 (1), V499 (13), V580 (1), V719 (1), V785 (1), V878 (7), V892 (10), V899 (4)
Other external causes of death (*n*=200)	S720 (1), S729 (2), T07X (1), T874 (1), W018 (1), W060 (1), W100 (14), W104 (1), W105 (1), W108 (2), W126 (1), W130 (11), W134 (3), W135 (1), W138 (1), W139 (2), W170 (4), W172 (1), W174 (1), W176 (1), W178 (2), W179 (1), W18 (1), W180 (3), W181 (1), W184 (3), W188 (1), W190 (13), W194 (2), W195 (1), W199 (2), W200 (1), W206 (1), W250 (1), W314 (1), W340 (1), W370 (1), W704 (1), W744 (1), W748 (3), W769 (1), W780 (1), W789 (1), W849 (2), W871 (1), W878 (2), X09 (1), X090 (3), X094 (1), X099 (1), X219 (1), X360 (1), X470 (1), X590 (3), X594 (1), X598 (1), X599 (30), Y200 (1), Y240 (1), Y244 (4), Y245 (1), Y248 (1), Y249 (2), Y260 (2), Y280 (1), Y330 (1), Y334 (1), Y338 (1), Y340 (5), Y344 (6), Y346 (1), Y348 (2), Y349 (16), Y405 (1), Y579 (2), Y838 (1), Y839 (4), Y841 (1), Y846 (1)
Liver cancer (*n*=200)	C220 (57), C221 (27), C229 (116)
Other liver disease (*n*=961)	B169 (2), B171 (23), B181 (2), B182 (13), B189 (1), B190 (2), B199 (2), I850 (17), I859 (5), K701 (19), K702 (1), K703 (161), K704 (26), K709 (19), K711 (2), K716 (1), K720 (12), K721 (70), K729 (216), K739 (2), K742 (1), K743 (2), K745 (2), K746 (291), K750 (17), K754 (3), K759 (2), K761 (1), K764 (1), K766 (13), K767 (14), K768 (1), K769 (17)
Upper aerodigestive cancer (*n*=58)	C029 (12), C049 (1), C069 (1), C07X (2), C089 (1), C109 (3), C119 (3), C139 (1), C140 (4), C142 (1), C159 (17), C329 (12)
Tuberculosis (*n*=21)	A162 (9), A165 (1), A169 (2), A170 (1), A180 (1), A181 (1), A182 (1), A183 (1), A199 (3), B909 (1)
Pneumonia (*n*=997)	J069 (1), J09 (3), J09X (1), J100 (2), J110 (1), J111 (1), J129 (4), J151 (3), J157 (2), J159 (42), J180 (57), J181 (34), J182 (2), J188 (1), J189 (643), J209 (3), J22X (20), J348 (1), J391 (1), J60X (1), J64X (5), J677 (1), J679 (2), J680 (1), J690 (4), J80X (4), J81X (5), J841 (82), J849 (8), J850 (2), J852 (2), J860 (1), J869 (7), J90X (5), J939 (1), J942 (2), J960 (5), J961 (1), J969 (3), J980 (1), J981 (1), J984 (17), J985 (5), J988 (7), J989 (2)
Non-MI acute IHD (*n*=37)	I249 (37)
Non-neoplastic pancreatic disease (*n*=82)	K851 (1), K852 (4), K858 (7), K859 (38), K85X (25), K861 (3), K868 (4)
Ill-specified disease (*n*=309)	R040 (1), R042 (1), R100 (3), R11X (1), R190 (1), R568 (1), R570 (35), R571 (16), R572 (5), R578 (1), R579 (2), R58X (3), R64X (1), R688 (23), R69X (1), R99X (214)

* The full list of ICD-10 codes pre-specified as potentially alcohol related (taken from page 10 of the appendix of reference 16) is: alcohol poisoning (F10, T51, X45, Y15); suicide (X60-84); assault (X85-Y09, Y35-36); transport accidents (V00-99); other external (remainder of S00-T99, W00-Y99); liver cancer (C22); other liver disease (B15-19, K70-77, I85); upper aerodigestive cancer (C00-15, C32); tuberculosis (A15-19, B90); pneumonia (J00-39, J60-98); non-MI acute IHD (I24); Non-neoplastic pancreatic disease (K85-86); and Ill-specified diseases (R00-99). Only the subset of these codes actually identified as *primary* causes of death at ages 35-74 years in the MCPS study population are listed in the table above.

Webtable 2b. ICD-10 codes of the causes of death not pre-specified as potentially alcohol-related

Underlying cause	ICD-10 codes (number of deaths at ages 35-74 years)*
Cardiac (*n*=2464)	I018 (1), I050 (2), I051 (2), I059 (14), I070 (1), I071 (2), I080 (3), I081 (1), I091 (1), I099 (16), I110 (104), I119 (11), I200 (4), I209 (3), I210 (19), I211 (9), I219 (1850), I220 (2), I221 (1), I229 (1), I251 (35), I252 (1), I258 (9), I259 (87), I270 (7), I272 (3), I279 (8), I301 (1), I319 (1), I330 (7), I340 (4), I348 (1), I350 (12), I351 (1), I358 (1), I38X (9), I420 (11), I426 (1), I429 (2), I442 (6), I443 (2), I460 (2), I461 (1), I469 (10), I471 (2), I472 (2), I489 (4), I48X (7), I490 (5), I499 (7), I500 (57), I501 (12), I509 (78), I515 (1), I517 (1), I518 (4), I519 (10), Q210 (1), Q231 (1), Q238 (1), Q248 (2)
Stroke (*n*=763)	F019 (2), I600 (2), I602 (1), I608 (1), I609 (80), I61 (1), I610 (1), I612 (2), I613 (1), I614 (2), I615 (2), I618 (1), I619 (224), I620 (9), I629 (6), I633 (4), I634 (18), I635 (4), I638 (2), I639 (54), I64X (105), I669 (5), I671 (6), I672 (1), I673 (1), I674 (4), I678 (83), I679 (101), I690 (1), I693 (7), I694 (9), I698 (23)
Other vascular (*n*=298)	E115 (37), E145 (34), I260 (2), I269 (82), I710 (4), I712 (1), I713 (5), I718 (3), I719 (1), I729 (2), I739 (3), I740 (1), I741 (1), I743 (1), I771 (14), I776 (1), I802 (7), I803 (1), I822 (1), I828 (1), I829 (3), I830 (1), I872 (7), I879 (1), I890 (1), I99X (4), K550 (70), K551 (1), K552 (1), K559 (7)
Cancer (*n*=1947)	C160 (3), C169 (212), C170 (12), C179 (1), C182 (3), C184 (1), C187 (2), C189 (125), C19X (4), C20X (19), C211 (1), C23X (28), C240 (12), C241 (9), C248 (3), C249 (15), C250 (24), C259 (100), C260 (3), C269 (3), C319 (1), C33X (1), C349 (154), C37X (1), C382 (1), C383 (1), C384 (1), C402 (1), C412 (2), C414 (1), C419 (7), C435 (1), C437 (1), C438 (1), C439 (15), C444 (1), C445 (1), C447 (1), C449 (6), C450 (1), C451 (2), C457 (1), C459 (4), C469 (1), C479 (1), C480 (10), C482 (5), C492 (3), C495 (1), C499 (12), C509 (204), C519 (4), C52X (1), C530 (1), C539 (112), C541 (27), C549 (2), C55X (11), C56X (111), C609 (2), C61X (69), C629 (1), C64X (87), C679 (23), C680 (3), C689 (1), C694 (1), C709 (2), C710 (14), C711 (1), C718 (2), C719 (41), C720 (1), C729 (1), C73X (26), C741 (1), C749 (1), C759 (1), C760 (6), C762 (3), C763 (2), C764 (1), C765 (1), C779 (1), C780 (6), C786 (5), C787 (8), C788 (2), C793 (2), C794 (3), C795 (1), C796 (1), C798 (3), C800 (29), C809 (22), C80X (3), C817 (1), C819 (9), C829 (1), C830 (1), C833 (13), C838 (1), C839 (2), C844 (1), C845 (2), C851 (2), C857 (1), C859 (48), C900 (47), C901 (1), C902 (1), C910 (26), C911 (1), C919 (2), C920 (30), C921 (7), C927 (3), C929 (3), C950 (2), C959 (3), C97X (1), D371 (6), D372 (1), D374 (5), D376 (6), D377 (2), D380 (2), D381 (7), D383 (2), D391 (4), D397 (1), D410 (3), D419 (1), D429 (1), D430 (13), D432 (2), D449 (1), D486 (1), D487 (9), D489 (2)
Renal (*n*=2156)	E102 (12), E112 (902), E122 (1), E142 (347), I120 (111), I129 (2), I130 (2), I131 (2), I132 (28), N002 (1), N009 (7), N039 (25), N049 (1), N059 (10), N10X (2), N119 (1), N12X (12), N142 (1), N151 (14), N179 (81), N180 (13), N185 (32), N189 (238), N19X (65), N200 (6), N201 (1), N289 (4), N300 (1), N309 (1), N390 (232), Q619 (1)
COVID-19 (*n*=895)	U071 (568), U072 (327)
Other respiratory (*n*=333)	B206 (2), B441 (1), E848 (1), J42X (14), J439 (31), J440 (92), J441 (2), J448 (6), J449 (165), J459 (12), J46X (3), J47X (2), Q311 (1), U099 (1)
Gastrointestinal (*n*=553)	A047 (3), A060 (1), A090 (21), A099 (34), A09X (13), B462 (1), K052 (1), K088 (1), K102 (1), K137 (1), K222 (1), K223 (2), K228 (2), K251 (3), K254 (9), K255 (12), K256 (3), K259 (6), K264 (7), K265 (3), K269 (2), K274 (3), K275 (1), K290 (6), K291 (2), K292 (1), K295 (4), K297 (1), K318 (7), K352 (5), K353 (3), K358 (5), K359 (2), K37X (1), K389 (1), K403 (1), K404 (1), K409 (1), K419 (1), K420 (4), K421 (1), K429 (2), K430 (2), K439 (1), K440 (1), K460 (4), K461 (1), K469 (4), K513 (1), K519 (1), K529 (6), K560 (2), K562 (1), K566 (40), K572 (2), K578 (8), K579 (12), K593 (5), K610 (4), K611 (1), K612 (1), K626 (1), K628 (1), K630 (1), K631 (22), K632 (4), K635 (1), K638 (2), K639 (4), K650 (29), K658 (1), K659 (56), K918 (1), K920 (30), K921 (3), K922 (119), K931 (1)
Hepatobiliary (*n*=84)	D136 (1), I81X (1), K563 (1), K800 (4), K801 (10), K802 (5), K803 (4), K804 (1), K805 (2), K810 (16), K811 (4), K819 (5), K821 (1), K822 (2), K829 (6), K830 (16), K831 (3), K839 (1), Q447 (1)
Acute diabetic (*n*=581)	E100 (4), E101 (4), E110 (170), E111 (192), E140 (109), E141 (94), E162 (8)
Other causes (*n*=748)	A415 (1), A419 (201), A480 (1), A483 (1), A498 (1), A810 (2), A86X (2), B200 (1), B201 (1), B207 (4), B208 (9), B210 (1), B212 (1), B218 (1), B227 (2), B238 (6), B24X (5), B259 (1), B451 (1), B465 (2), B690 (2), B948 (2), B99X (1), D033 (1), D27X (1), D320 (1), D329 (9), D352 (1), D361 (1), D420 (1), D464 (1), D467 (1), D469 (7), D471 (1), D472 (1), D474 (1), D479 (1), D619 (6), D649 (6), D682 (1), D693 (3), D694 (1), D696 (3), D699 (3), D70X (2), D733 (1), D739 (1), D762 (1), E035 (2), E039 (17), E049 (1), E055 (1), E059 (3), E065 (1), E116 (2), E119 (5), E129 (1), E146 (4), E149 (9), E230 (1), E249 (2), E279 (1), E43X (2), E440 (1), E660 (1), E725 (1), E835 (1), E86X (5), E870 (1), E872 (13), E875 (2), E876 (1), E878 (1), E889 (2), F03X (6), F09X (1), F182 (1), F209 (2), G009 (3), G039 (4), G042 (2), G049 (8), G060 (2), G10X (6), G121 (1), G122 (21), G20X (9), G231 (1), G300 (1), G301 (1), G309 (5), G310 (1), G35X (3), G379 (1), G403 (1), G409 (16), G419 (2), G439 (1), G589 (1), G610 (5), G629 (1), G709 (1), G710 (3), G712 (1), G809 (1), G819 (1), G822 (1), G919 (4), G931 (11), G934 (5), G935 (1), G936 (2), G958 (1), G959 (1), H440 (1), H669 (1), I10X (3), L021 (4), L022 (6), L023 (2), L024 (1), L031 (5), L038 (1), L039 (4), L089 (45), L100 (1), L109 (1), L511 (1), L512 (1), L899 (8), L89X (5), L905 (1), L921 (1), L958 (1), L984 (7), L988 (1), M009 (1), M050 (1), M068 (1), M069 (21), M100 (1), M109 (1), M139 (1), M199 (1), M311 (1), M313 (1), M319 (1), M321 (4), M329 (2), M340 (1), M348 (1), M349 (1), M350 (1), M469 (1), M600 (2), M623 (7), M725 (1), M726 (18), M798 (23), M799 (1), M844 (1), M869 (3), M993 (1), N40X (6), N410 (1), N498 (9), N499 (1), N719 (1), N739 (2), N764 (1), N948 (1), O720 (1)

* Only codes identified as *primary* causes of death at ages 35-74 years in the MCPS study population are listed.

Webtable 3. Self-reported baseline alcohol consumption among 21 136 'regular' drinkers

Preferred alcohol product, among regular drinkers	Regular drinkers (grams/day) *				
	<70 (*n*=12 188)	≥70 to <140 (*n*=3677)	≥140 to <210 (*n*=2057)	≥210 (*n*=3214)	All regular (*n*=21 136)
Beer					
n (column %)	3547 (29%)	1221 (33%)	723 (35%)	1089 (34%)	6580 (31%)
Weekly alcohol amount (g), mean (SD)	26 (15)	88 (7)	153 (14)	378 (226)	110 (157)
Weekly number of glasses, mean (SD)	1.6 (0.9)	5.4 (0.4)	9.3 (0.9)	22.9 (13.7)	6.7 (9.5)
Wine					
n (column %)	293 (2%)	46 (1%)	77 (4%)	15 (<0.5%)	431 (2%)
Weekly alcohol amount (g), mean (SD)	23 (15)	88 (4)	154 (12)	458 (285)	68 (104)
Weekly number of glasses, mean (SD)	1.3 (0.9)	5.2 (0.2)	9.2 (0.7)	27.2 (17.0)	4.1 (6.2)
Spirits					
n (column %)	8096 (66%)	2275 (62%)	1219 (59%)	1793 (56%)	13 383 (63%)
Weekly alcohol amount (g), mean (SD)	26 (14)	85 (7)	149 (16)	386 (253)	96 (153)
Weekly number of glasses, mean (SD)	1.6 (0.9)	5.3 (0.5)	9.3 (1.0)	24.2 (15.8)	6.0 (9.5)
Higher alcohol percentage products †					
n (column %)	23 (<0.5%)	29 (1%)	5 (<0.5%)	92 (3%)	149 (1%)
Weekly alcohol amount (g), mean (SD)	33 (14)	104 (26)	184 (0)	767 (360)	505 (439)
Weekly number of glasses, mean (SD)	2.2 (0.9)	6.9 (1.7)	12.3 (0.0)	51.1 (24.0)	33.7 (29.2)
Other (e.g. cooler, pulque)					
n (column %)	229 (2%)	106 (3%)	33 (2%)	225 (7%)	593 (3%)
Weekly alcohol amount (g), mean (SD)	31 (19)	107 (26)	177 (22)	602 (449)	270 (382)
Weekly number of glasses, mean (SD)	1.5 (1.0)	5.9 (1.8)	11.5 (2.7)	31.1 (22.5)	14.1 (19.4)

* Regular drinkers are those who reported drinking alcohol at least on a monthly basis. Those reporting to drink at least monthly but less than weekly are included in the <70g/week category. † Listed as 'pure alcohol' in the baseline questionnaire (assumed to corespond to products with alcohol percentages higher than spirits).

Webtable 4a. Baseline characteristics of 45 065 men aged 35-74 years by self-reported alcohol consumption

	Never drinker (*n*=2 650)	Former drinker (*n*=7162)	Occasional drinker (*n*=20 390)	Regular drinkers (grams / week)				
				<70 (*n*=7231)	≥70 to <140 (*n*=3026)	≥140 to <210 (*n*=1617)	≥210 (*n*=2989)	All regular (*n*=14 863)
Alcohol consumption								
Weekly amount (g)	-	-	-	28 (14-39)	86 (79-96)	144 (144-149)	288 (235-347)	76 (29-149)
Preferred alcohol product								
Beer	-	1552 (22%)	5030 (25%)	2355 (33%)	1054 (35%)	594 (37%)	1026 (34%)	5029 (34%)
Wine	-	76 (1%)	565 (3%)	106 (1%)	32 (1%)	45 (3%)	13 (<0.5%)	196 (1%)
Spirits	-	4978 (70%)	14 184 (70%)	4637 (64%)	1831 (61%)	946 (59%)	1679 (56%)	9093 (61%)
Higher alcohol percentage products *	-	167 (2%)	67 (<0.5%)	21 (<0.5%)	25 (1%)	4 (<0.5%)	88 (3%)	138 (1%)
Other (e.g. cooler, pulque)	-	331 (5%)	535 (3%)	112 (2%)	84 (3%)	28 (2%)	183 (6%)	407 (3%)
Age, years	51.5 (11.5)	54.4 (11.3)	50.9 (10.8)	49.3 (10.2)	49.0 (10.2)	51.1 (10.7)	49.1 (10.1)	49.4 (10.3)
Resident of Coyoacán	845 (32%)	2847 (40%)	8258 (41%)	3759 (52%)	1590 (53%)	882 (55%)	1492 (50%)	7723 (52%)
University or college educated	720 (27%)	1031 (14%)	5162 (25%)	2542 (35%)	859 (28%)	526 (33%)	545 (18%)	4472 (30%)
Smoking behaviour								
Never smoker	1287 (49%)	1146 (16%)	4376 (21%)	1374 (19%)	408 (13%)	244 (15%)	356 (12%)	2382 (16%)
Ex-smoker	644 (24%)	2730 (38%)	5730 (28%)	1914 (26%)	700 (23%)	393 (24%)	594 (20%)	3601 (24%)
Current smoker	719 (27%)	3286 (46%)	10 284 (50%)	3943 (55%)	1918 (63%)	980 (61%)	2039 (68%)	8880 (60%)
Regular leisure-time physical activity †	775 (29%)	1874 (26%)	6078 (30%)	2529 (35%)	980 (32%)	549 (34%)	806 (27%)	4864 (33%)
Previously-diagnosed diabetes	366 (14%)	1382 (19%)	2564 (13%)	711 (10%)	284 (9%)	163 (10%)	316 (11%)	1474 (10%)
Physical measurements								
Body mass index, kg/m²	27.9 (4.7)	28.0 (4.4)	28.2 (4.4)	27.9 (4.1)	27.9 (4.2)	27.6 (4.1)	27.7 (4.6)	27.8 (4.2)
Waist-hip ratio	0.95 (0.07)	0.96 (0.07)	0.95 (0.06)	0.95 (0.07)	0.95 (0.06)	0.95 (0.06)	0.95 (0.06)	0.95 (0.06)
Systolic blood pressure, mmHg	127.7 (15.6)	129.3 (16.5)	127.7 (15.2)	127.1 (14.8)	127.4 (14.4)	129.0 (15.4)	128.8 (15.3)	127.7 (14.9)
Diastolic blood pressure, mmHg	83.8 (9.7)	84.5 (10.0)	84.3 (9.8)	84.2 (9.6)	84.5 (9.6)	85.0 (10.0)	85.2 (10.0)	84.6 (9.8)
Laboratory measurements ‡								
LDL cholesterol, mmol/l	2.40 (0.77)	2.34 (0.77)	2.40 (0.78)	2.45 (0.81)	2.44 (0.78)	2.43 (0.80)	2.34 (0.82)	2.42 (0.80)
HDL cholesterol, mmol/l	0.92 (0.17)	0.91 (0.17)	0.92 (0.18)	0.94 (0.19)	0.97 (0.20)	0.98 (0.21)	1.01 (0.25)	0.96 (0.21)
Triglycerides, mmol/l	1.57 (0.61)	1.60 (0.65)	1.66 (0.66)	1.67 (0.69)	1.72 (0.72)	1.77 (0.75)	1.79 (0.81)	1.71 (0.73)
Apolipoprotein A1, g/l	1.15 (0.16)	1.13 (0.17)	1.16 (0.17)	1.18 (0.18)	1.21 (0.19)	1.23 (0.20)	1.25 (0.23)	1.20 (0.20)
Apolipoprotein B, g/l	0.88 (0.20)	0.87 (0.20)	0.89 (0.20)	0.90 (0.21)	0.89 (0.20)	0.89 (0.21)	0.88 (0.22)	0.89 (0.21)

Results shown are n (column %), mean (standard deviation) or median (interquartile range). Occasional drinkers are those who reported drinking alcohol on a less than monthly basis. Regular drinkers are those reporting drinking at least monthly. Those reporting to drink monthly but less than weekly are included in the <70g/week category. For reference, 140g pure alcohol is about equivalent to 3L beer, 1.5 bottles of wine or 350ml spirits. * Listed as 'pure alcohol' in the baseline questionnaire (assumed to correspond to products with alcohol percentages higher than spirits). † At least 1 day/week. ‡ Measured using the Nightingale Health NMR platform.

Webtable 4b. Baseline characteristics of 93 348 women aged 35-74 years by self-reported alcohol consumption

	Never drinker (*n*=23 894)	Former drinker (*n*=10 467)	Occasional drinker (*n*=52 714)	Regular drinkers (grams / week)				
				<70 (*n*=4957)	≥70 to <140 (*n*=651)	≥140 to <210 (*n*=440)	≥210 (*n*=225)	All regular (*n*=6273)
Alcohol consumption								
Weekly amount (g)	-	-	-	14 (12-36)	84 (84-87)	144 (144-149)	288 (228-347)	28 (12-48)
Preferred alcohol product								
Beer	-	1888 (18%)	9421 (18%)	1192 (24%)	167 (26%)	129 (29%)	63 (28%)	1551 (25%)
Wine	-	586 (6%)	5654 (11%)	187 (4%)	14 (2%)	32 (7%)	2 (1%)	235 (4%)
Spirits	-	7061 (67%)	33 731 (64%)	3459 (70%)	444 (68%)	273 (62%)	114 (51%)	4290 (68%)
Higher alcohol percentage products *	-	41 (<0.5%)	44 (<0.5%)	2 (<0.5%)	4 (1%)	1 (<0.5%)	4 (2%)	11 (<0.5%)
Other (e.g. cooler, pulque)	-	718 (7%)	3812 (7%)	117 (2%)	22 (3%)	5 (1%)	42 (19%)	186 (3%)
Age, years	51.3 (11.4)	52.4 (11.1)	49.4 (10.3)	48.2 (9.7)	48.3 (9.5)	50.5 (10.9)	48.4 (10.3)	48.4 (9.8)
Resident of Coyoacán	7547 (32%)	3805 (36%)	20 688 (39%)	3018 (61%)	372 (57%)	255 (58%)	101 (45%)	3746 (60%)
University or college educated	2253 (9%)	820 (8%)	6840 (13%)	1291 (26%)	141 (22%)	84 (19%)	20 (9%)	1536 (24%)
Smoking behaviour								
Never smoker	18 986 (79%)	5928 (57%)	30 607 (58%)	1959 (40%)	181 (28%)	144 (33%)	73 (32%)	2357 (38%)
Ex-smoker	2215 (9%)	2214 (21%)	7710 (15%)	857 (17%)	121 (19%)	63 (14%)	23 (10%)	1064 (17%)
Current smoker	2693 (11%)	2325 (22%)	14 397 (27%)	2141 (43%)	349 (54%)	233 (53%)	129 (57%)	2852 (45%)
Regular leisure-time physical activity †	3642 (15%)	1652 (16%)	10 371 (20%)	1477 (30%)	175 (27%)	128 (29%)	34 (15%)	1814 (29%)
Previously-diagnosed diabetes	3595 (15%)	2009 (19%)	5788 (11%)	307 (6%)	44 (7%)	26 (6%)	25 (11%)	402 (6%)
Physical measurements								
Body mass index, kg/m²	29.7 (5.3)	30.0 (5.4)	29.7 (5.2)	28.5 (4.9)	28.8 (5.1)	28.3 (5.1)	29.2 (5.4)	28.6 (5.0)
Waist-hip ratio	0.88 (0.07)	0.89 (0.07)	0.87 (0.07)	0.86 (0.07)	0.86 (0.08)	0.86 (0.07)	0.88 (0.08)	0.86 (0.07)
Systolic blood pressure, mmHg	127.6 (17.4)	127.9 (17.4)	125.2 (16.0)	123.1 (15.1)	123.5 (15.6)	124.6 (15.9)	126.8 (16.5)	123.4 (15.2)
Diastolic blood pressure, mmHg	82.9 (10.4)	83.0 (10.3)	82.0 (10.0)	81.0 (9.8)	81.0 (9.6)	81.6 (10.1)	82.5 (9.8)	81.1 (9.8)
Laboratory measurements ‡								
LDL cholesterol, mmol/l	2.45 (0.79)	2.46 (0.82)	2.49 (0.78)	2.58 (0.76)	2.59 (0.81)	2.59 (0.82)	2.44 (0.92)	2.58 (0.77)
HDL cholesterol, mmol/l	1.03 (0.21)	1.02 (0.21)	1.03 (0.21)	1.07 (0.23)	1.10 (0.25)	1.12 (0.24)	1.06 (0.28)	1.08 (0.23)
Triglycerides, mmol/l	1.55 (0.66)	1.57 (0.67)	1.53 (0.64)	1.47 (0.61)	1.49 (0.66)	1.48 (0.58)	1.64 (0.70)	1.48 (0.62)
Apolipoprotein A1, g/l	1.24 (0.19)	1.24 (0.20)	1.25 (0.19)	1.27 (0.20)	1.30 (0.22)	1.31 (0.21)	1.29 (0.25)	1.28 (0.20)
Apolipoprotein B, g/l	0.89 (0.21)	0.90 (0.22)	0.90 (0.21)	0.91 (0.21)	0.91 (0.22)	0.91 (0.23)	0.90 (0.26)	0.91 (0.21)

Results shown are n (column %), mean (standard deviation) or median (interquartile range). Occasional drinkers are those who reported drinking alcohol on a less than monthly basis. Regular drinkers are those reporting drinking at least monthly. Those reporting to drink monthly but less than weekly are included in the <70g/week category. For reference, 140g pure alcohol is about equivalent to 3L beer, 1.5 bottles of wine or 350ml spirits. * Listed as 'pure alcohol' in the baseline questionnaire (assumed to correspond to products with alcohol percentages higher than spirits). † At least 1 day/week. ‡ Measured using the Nightingale Health NMR platform.

Webtable 5. Additional baseline characteristics of participants aged 35-74 years by self-reported alcohol consumption

	Never drinker	Former drinker	Occasional drinker *	Regular drinkers (grams / week) †				
				<70	≥70 to <140	≥140 to <210	≥210	All regular
	(*n*=26 537)	(*n*=17 622)	(*n*=73 097)	(*n*=12 185)	(*n*=3677)	(*n*=2057)	(*n*=3212)	(*n*=21 131)
Income among men (pesos/month) ‡								
Number of men	2650 (10%)	7159 (41%)	20 388 (28%)	7228 (59%)	3026 (82%)	1617 (79%)	2987 (93%)	14 858 (70%)
Zero income reported	489 (18%)	1114 (16%)	2058 (10%)	550 (8%)	235 (8%)	103 (6%)	286 (10%)	1174 (8%)
>0 to <1500	545 (21%)	1801 (25%)	3734 (18%)	1093 (15%)	484 (16%)	284 (18%)	637 (21%)	2498 (17%)
≥1500 to <3000	642 (24%)	2025 (28%)	5660 (28%)	1780 (25%)	815 (27%)	409 (25%)	915 (31%)	3919 (26%)
≥3000 to <5000	482 (18%)	1313 (18%)	4577 (22%)	1590 (22%)	682 (23%)	330 (20%)	634 (21%)	3236 (22%)
≥5000	492 (19%)	906 (13%)	4359 (21%)	2215 (31%)	810 (27%)	491 (30%)	515 (17%)	4031 (27%)
Consumption of fruit and vegetables ≥3 days/week	14 392 (54%)	9033 (51%)	39 810 (54%)	6769 (56%)	1744 (47%)	1055 (51%)	1284 (40%)	10 852 (51%)
Previously-diagnosed hypertension	6031 (23%)	4136 (23%)	13 089 (18%)	1513 (12%)	382 (10%)	267 (13%)	319 (10%)	2481 (12%)
Medication use								
Anti-hypertensive	4449 (17%)	3111 (18%)	9324 (13%)	1079 (9%)	229 (6%)	184 (9%)	193 (6%)	1685 (8%)
Lipid-lowering	105 (<0.5%)	112 (1%)	379 (1%)	68 (1%)	16 (<0.5%)	7 (<0.5%)	10 (<0.5%)	101 (<0.5%)
Anti-thrombotic	617 (2%)	521 (3%)	1694 (2%)	256 (2%)	59 (2%)	47 (2%)	45 (1%)	407 (2%)
Respiratory $	520 (2%)	383 (2%)	1277 (2%)	178 (1%)	34 (1%)	33 (2%)	33 (1%)	278 (1%)

Results shown are *n* (column %) or, for income in men, *n* (% of men). The number of participants in each group differs slightly from the numbers in Table 1 because they exclude the few (*n*=26) with missing data on fruit and vegetables consumption. *Occasional drinkers are those who reported drinking alcohol on a less than monthly basis. † Regular drinkers are those reporting drinking at least monthly. Those reporting to drink monthly but less than weekly are included in the <70g/week category. ‡ Men only since 64% of women reported no income. $ Includes inhaled bronchodilators, inhaled corticosteroids, and other respiratory drugs.

Webtable 6. Self-reported alcohol consumption at baseline and at resurvey ~15 years later

Baseline-defined alcohol consumption category	No. participants (*n*=9885)	Resurvey-defined alcohol consumption category				
			Former		Current	
		Never (*n*=2833)	Gave up because of ill health (*n*=402)	Did not give up because of ill health (*n*=1502)	Less than weekly (*n*=4809)	At least weekly (*n*=339)
Never drinker	1922	1191 (61%)	30 (2%)	206 (10%)	486 (25%)	9 (<0.5%)
Former drinker	1251	344 (27%)	105 (8%)	305 (24%)	470 (36%)	27 (2%)
Occasional drinker *	5297	1180 (22%)	178 (3%)	818 (15%)	3006 (55%)	115 (2%)
Regular drinker (g/week) †						
<70	842	89 (10%)	39 (5%)	95 (11%)	548 (63%)	71 (8%)
≥70 to <140	225	7 (3%)	15 (6%)	27 (12%)	133 (58%)	43 (19%)
≥140 to <210	127	11 (9%)	14 (11%)	21 (16%)	60 (47%)	21 (16%)
≥210	221	11 (5%)	21 (9%)	30 (13%)	106 (47%)	53 (23%)

Percentages shown are row percentages. Analyses are limited to 9885 participants with non-missing data on alcohol consumption at resurvey. *Occasional drinkers are those who reported drinking alcohol on a less than monthly basis. † Regular drinkers are those reporting drinking at least monthly. Those reporting to drink monthly but less than weekly are included in the <70g/week category.

Webtable 7. Alcohol consumption versus all-cause and alcohol-related mortality at ages 35-74 years, by sex

		All deaths		Alcohol-related causes	
	**No. participants**	**No. deaths**	**RR (95% CI)**	**No. deaths**	**RR (95% CI)**
Men					
Never	2650	352	1.09 (0.97, 1.22)	75	1.02 (0.80, 1.31)
Former	7162	1228	1.22 (1.14, 1.30)	254	1.21 (1.04, 1.41)
Occasional *	20 390	2600	1.00	571	1.00
Regular (grams/week) †					
<70	7231	775	0.92 (0.85, 1.00)	202	1.06 (0.90, 1.25)
≥70 to <140	3026	381	1.07 (0.96, 1.20)	134	1.65 (1.37, 2.00)
≥140 to <210	1617	206	1.09 (0.94, 1.25)	70	1.65 (1.29, 2.12)
≥210	2989	524	1.43 (1.30, 1.57)	229	2.73 (2.34, 3.19)
Women					
Never	23 894	2245	1.16 (1.10, 1.22)	402	1.04 (0.92, 1.17)
Former	10 467	1145	1.20 (1.12, 1.28)	225	1.21 (1.04, 1.40)
Occasional *	52 714	4056	1.00	813	1.00
Regular (grams/week) †					
<70	4957	268	0.85 (0.75, 0.97)	57	0.87 (0.66, 1.14)
≥70 to <140	651	57	1.32 (1.02, 1.72)	17	1.91 (1.18, 3.09)
≥140 to <210	440	31	1.08 (0.76, 1.54)	9	1.52 (0.79, 2.94)
≥210	225	21	1.24 (0.81, 1.90)	9	2.68 (1.39, 5.18)

Analyses are stratified by age-at-risk and district and adjusted for education, smoking status, leisure-time physical activity and self-reported diabetes at baseline. *Occasional drinkers are those who reported drinking alcohol on a less than monthly basis. † Regular drinkers are those who reported drinking alcohol at least on a monthly basis. Those reporting to drink at least monthly but less than weekly are included in the <70g/week category.

Webtable 8. Sensitivity analyses

	No. participants	All deaths: No. deaths	All deaths: RR (95% CI)	Alcohol-related causes: No. deaths	Alcohol-related causes: RR (95% CI)
Limited to never smokers					
Never	20 273	1908	1.13 (1.06, 1.19)	359	1.03 (0.91, 1.18)
Former	7074	776	1.12 (1.04, 1.21)	168	1.17 (0.99, 1.39)
Occasional *	34 983	2926	1.00	618	1.00
Regular (grams/week) †					
<70	3333	236	0.84 (0.73, 0.96)	45	0.68 (0.50, 0.93)
≥70 to <140	589	69	1.21 (0.95, 1.54)	26	1.85 (1.24, 2.77)
≥140 to <210	388	44	1.17 (0.86, 1.58)	15	1.65 (0.98, 2.77)
≥210	429	86	1.82 (1.46, 2.26)	43	3.67 (2.65, 5.09)
Limited to those without diabetes					
Never	22 583	1610	1.15 (1.09, 1.22)	321	0.99 (0.87, 1.12)
Former	14 238	1356	1.21 (1.13, 1.28)	317	1.19 (1.05, 1.35)
Occasional *	64 752	4473	1.00	1048	1.00
Regular (grams/week) †					
<70	11 170	749	0.89 (0.82, 0.96)	200	0.97 (0.83, 1.13)
≥70 to <140	3349	337	1.16 (1.04, 1.30)	127	1.73 (1.43, 2.09)
≥140 to <210	1868	185	1.16 (1.00, 1.34)	68	1.71 (1.34, 2.20)
≥210	2873	415	1.54 (1.39, 1.71)	209	3.02 (2.58, 3.54)
Limited to never smokers without diabetes					
Never	17 239	1172	1.13 (1.05, 1.21)	234	0.96 (0.82, 1.13)
Former	5683	456	1.20 (1.08, 1.33)	118	1.27 (1.04, 1.56)
Occasional *	30 922	1953	1.00	463	1.00
Regular (grams/week) †					
<70	3067	186	0.91 (0.78, 1.06)	43	0.80 (0.58, 1.10)
≥70 to <140	542	50	1.21 (0.91, 1.61)	25	2.10 (1.39, 3.17)
≥140 to <210	347	33	1.26 (0.89, 1.78)	12	1.62 (0.91, 2.89)
≥210	374	60	1.95 (1.50, 2.54)	34	3.64 (2.52, 5.26)
Including those with prior disease					
Never	27 891	2872	1.16 (1.11, 1.21)	521	1.06 (0.96, 1.18)
Former	19 208	2834	1.28 (1.22, 1.33)	569	1.32 (1.19, 1.45)
Occasional *	76 011	7178	1.00	1456	1.00
Regular (grams/week) †					
<70	12 631	1108	0.89 (0.83, 0.95)	270	1.01 (0.88, 1.15)
≥70 to <140	3790	462	1.09 (0.99, 1.21)	155	1.67 (1.41, 1.99)
≥140 to <210	2139	253	1.08 (0.95, 1.22)	82	1.61 (1.28, 2.01)
≥210	3325	571	1.40 (1.28, 1.53)	247	2.76 (2.40, 3.19)
Extending age-at-risk to 35-89 years					
Never	29 333	5248	1.11 (1.08, 1.15)	944	1.06 (0.98, 1.15)
Former	19 983	4886	1.16 (1.12, 1.20)	935	1.18 (1.09, 1.27)
Occasional *	76 630	11495	1.00	2249	1.00
Regular (grams/week) †					
<70	12 573	1710	0.92 (0.88, 0.97)	388	1.01 (0.90, 1.13)
≥70 to <140	3813	646	1.03 (0.95, 1.11)	190	1.42 (1.22, 1.65)
≥140 to <210	2227	425	1.05 (0.95, 1.16)	122	1.45 (1.21, 1.75)
≥210	3334	749	1.30 (1.20, 1.40)	296	2.38 (2.10, 2.70)
Excluding the first 5 years of follow-up					
Never	25 773	1952	1.11 (1.05, 1.17)	371	1.03 (0.91, 1.17)
Former	16 851	1691	1.15 (1.09, 1.22)	347	1.19 (1.05, 1.35)
Occasional *	71 658	5370	1.00	1107	1.00
Regular (grams/week) †					
<70	11 959	838	0.88 (0.82, 0.95)	192	0.94 (0.80, 1.10)

Webtable 8. Sensitivity analyses

	No. participants	All deaths: No. deaths	All deaths: RR (95% CI)	Alcohol-related causes: No. deaths	Alcohol-related causes: RR (95% CI)
≥70 to <140	3601	369	1.14 (1.02, 1.27)	119	1.68 (1.39, 2.05)
≥140 to <210	1994	176	1.01 (0.87, 1.17)	50	1.33 (1.00, 1.77)
≥210	3074	412	1.32 (1.19, 1.47)	158	2.36 (1.98, 2.81)
Extending the definition of alcohol-related causes to include further cancers ‡					
Never	26 544	2597	1.15 (1.10, 1.20)	751	1.04 (0.96, 1.14)
Former	17 629	2373	1.21 (1.15, 1.26)	656	1.11 (1.02, 1.22)
Occasional *	73 104	6656	1.00	2176	1.00
Regular (grams/week) †					
<70	12 188	1043	0.90 (0.85, 0.97)	365	0.92 (0.82, 1.03)
≥70 to <140	3677	438	1.11 (1.00, 1.22)	201	1.50 (1.29, 1.74)
≥140 to <210	2057	237	1.09 (0.96, 1.24)	103	1.39 (1.14, 1.70)
≥210	3214	545	1.43 (1.30, 1.56)	278	2.24 (1.96, 2.55)
Adjusting for additional covariates $					
Never	26 537	2597	1.14 (1.09, 1.19)	477	1.03 (0.92, 1.15)
Former	17 622	2369	1.18 (1.13, 1.24)	478	1.18 (1.06, 1.32)
Occasional *	73 097	6655	1.00	1384	1.00
Regular (grams/week) †					
<70	12 185	1043	0.92 (0.86, 0.98)	259	1.03 (0.90, 1.18)
≥70 to <140	3677	438	1.14 (1.03, 1.25)	151	1.74 (1.46, 2.07)
≥140 to <210	2057	237	1.12 (0.98, 1.27)	79	1.67 (1.32, 2.10)
≥210	3212	544	1.43 (1.30, 1.56)	238	2.75 (2.38, 3.19)

* Occasional drinkers are those who reported drinking alcohol on a less than monthly basis. † Regular drinkers are those who reported drinking alcohol at least on a monthly basis. Those reporting to drink at least monthly but less than weekly are included in the <70g/week category. ‡ Breast, oesophageal or stomach cancer. $ Additional covariates are self-reported income (zero, >0 to <1500, ≥1500 to <3000, ≥3000 to <5000, ≥5000 pesos/month), fruit and vegetable consumption (0, 1 to 2, 3 to 4, 5 to 7 days/week), previously-diagnosed hypertension, and antihypertensive, antithrombotic, lipid-lowering and respiratory medication use (Webtable 5).

Webtable 9. Alcohol consumption versus all-cause and alcohol-related mortality at ages 35-74 years, overall and by age-at-risk

	**35-74 years**			**35-54 years**			**55-74 years**		
	**No. participants**	**No. deaths**	**RR (95% CI)**	**No. participants**	**No. deaths**	**RR (95% CI)**	**No. participants**	**No. deaths**	**RR (95% CI)**
All deaths									
Never	26 544	2597	1.15 (1.10, 1.21)	16 929	431	1.40 (1.25, 1.58)	9615	2166	1.11 (1.05, 1.17)
Former	17 629	2373	1.20 (1.15, 1.26)	10 028	324	1.48 (1.30, 1.68)	7601	2049	1.17 (1.11, 1.23)
Occasional	73 104	6656	1.00	51 413	965	1.00	21 691	5691	1.00
Regular (<140 g/week)	15 865	1481	0.95 (0.90, 1.01)	11 667	289	1.23 (1.07, 1.41)	4198	1192	0.91 (0.85, 0.97)
Regular (≥140 g/week)	5271	782	1.30 (1.20, 1.40)	3708	194	2.09 (1.78, 2.47)	1563	588	1.15 (1.06, 1.26)
Alcohol-related causes									
Never	26 544	477	1.04 (0.94, 1.16)	16 929	76	1.07 (0.82, 1.40)	9615	401	1.03 (0.92, 1.16)
Former	17 629	479	1.21 (1.09, 1.34)	10 028	74	1.33 (1.03, 1.73)	7601	405	1.18 (1.05, 1.32)
Occasional	73 104	1384	1.00	51 413	244	1.00	21 691	1140	1.00
Regular (<140 g/week)	15 865	410	1.18 (1.05, 1.33)	11 667	109	1.53 (1.21, 1.94)	4198	301	1.10 (0.96, 1.25)
Regular (≥140 g/week)	5271	317	2.33 (2.05, 2.65)	3708	117	4.07 (3.20, 5.17)	1563	200	1.86 (1.59, 2.18)

Analyses are stratified by age-at-risk and district and adjusted for sex, education, smoking status, leisure-time physical activity and self-reported diabetes at baseline. Occasional drinkers are those who reported drinking alcohol on a less than monthly basis. Regular drinkers are those who reported drinking alcohol at least on a monthly basis. Those reporting to drink at least monthly but less than weekly are included in the <140g/week category.

Webtable 10. Alcohol consumption versus all-cause and alcohol-related mortality at ages 35-74 years, by period of follow-up

	First 10 years of follow-up			After the first 10 years of follow-up		
**Cause of death**	**No. participants**	**No. deaths**	**RR (95% CI)**	**No. participants**	**No. deaths**	**RR (95% CI)**
All deaths						
Never	26 544	1270	1.20 (1.12, 1.29)	24 787	1327	1.11 (1.04, 1.18)
Former	17 629	1300	1.34 (1.25, 1.43)	15 899	1073	1.09 (1.02, 1.17)
Occasional	73 104	2821	1.00	69 477	3835	1.00
Regular (<140 g/week)	15 865	595	0.97 (0.88, 1.06)	15 119	886	0.94 (0.87, 1.02)
Regular (≥140 g/week)	5271	383	1.55 (1.39, 1.73)	4826	399	1.12 (1.01, 1.25)
Alcohol-related causes						
Never	26 544	217	0.99 (0.84, 1.16)	24 787	260	1.09 (0.95, 1.27)
Former	17 629	258	1.24 (1.07, 1.44)	15 899	221	1.17 (1.01, 1.37)
Occasional	73 104	627	1.00	69 477	757	1.00
Regular (<140 g/week)	15 865	168	1.09 (0.91, 1.30)	15 119	242	1.26 (1.08, 1.47)
Regular (≥140 g/week)	5271	175	2.75 (2.30, 3.29)	4826	142	1.96 (1.62, 2.37)

Analyses are stratified by age-at-risk and district and adjusted for sex, education, smoking status, leisure-time physical activity and self-reported diabetes at baseline. Occasional drinkers are those who reported drinking alcohol on a less than monthly basis. Regular drinkers are those who reported drinking alcohol at least on a monthly basis. Those reporting to drink at least monthly but less than weekly are included in the <140g/week category.

Webtable 11. Alcohol product versus all-cause and alcohol-related mortality at ages 35-74 years in occasional and in regular drinkers, before and after adjustment for total alcohol consumed

		All deaths		Alcohol-related causes	
	**No. participants**	**No. deaths**	**RR (95% CI)**	**No. deaths**	**RR (95% CI)**
**Occasional drinkers** by preferred alcohol product					
Wine	6219	542	0.98 (0.89, 1.08)	96	0.89 (0.71, 1.12)
Beer	14 451	1478	1.00	312	1.00
Spirits	47 915	4213	0.90 (0.85, 0.96)	882	0.91 (0.80, 1.04)
Higher alcohol percentage products*	111	15	1.00 (0.60, 1.67)	5	1.56 (0.64, 3.77)
Other (e.g. cooler, pulque)	4347	390	0.98 (0.88, 1.10)	85	1.08 (0.85, 1.37)
**Occasional drinkers** by preferred alcohol product, further adjusted for total alcohol consumed					
Wine	6219	542	1.00 (0.90, 1.11)	96	0.92 (0.73, 1.17)
Beer	14 451	1478	1.00	312	1.00
Spirits	47 915	4213	0.90 (0.85, 0.96)	882	0.91 (0.80, 1.04)
Higher alcohol percentage products*	111	15	0.98 (0.59, 1.63)	5	1.50 (0.62, 3.63)
Other (e.g. cooler, pulque)	4347	390	0.99 (0.88, 1.11)	85	1.09 (0.86, 1.39)
**Regular drinkers** by preferred alcohol product					
Wine	431	30	0.84 (0.58, 1.21)	15	1.37 (0.81, 2.31)
Beer	6580	715	1.00	235	1.00
Spirits	13 383	1382	0.96 (0.87, 1.05)	412	0.89 (0.76, 1.05)
Higher alcohol percentage products*	149	38	2.19 (1.58, 3.03)	27	4.52 (3.03, 6.74)
Other (e.g. cooler, pulque)	593	98	1.40 (1.14, 1.73)	38	1.75 (1.24, 2.47)
**Regular drinkers** by preferred alcohol product, further adjusted for total alcohol consumed					
Wine	431	30	0.87 (0.60, 1.25)	15	1.44 (0.85, 2.43)
Beer	6580	715	1.00	235	1.00
Spirits	13 383	1382	0.96 (0.88, 1.05)	412	0.90 (0.76, 1.05)
Higher alcohol percentage products*	149	38	1.56 (1.12, 2.19)	27	2.23 (1.46, 3.40)
Other (e.g. cooler, pulque)	593	98	1.16 (0.93, 1.44)	38	1.09 (0.75, 1.57)

Analyses are stratified by age-at-risk and district and adjusted for sex, education, smoking status, leisure-time physical activity and self-reported diabetes at baseline. Occasional drinkers are those who reported drinking alcohol on a less than monthly basis. Regular drinkers are those who reported drinking alcohol at least on a monthly basis. * Listed as 'pure alcohol' in the baseline questionnaire (assumed to corespond to products with alcohol percentages higher than spirits).

Webtable 12. Alcohol consumption versus alcohol-related causes of death at ages 35-74 years, overall and by age-at-risk

	35-74 years			35-54 years			55-74 years		
**Cause of death, alcohol category**	**No. participants**	**No. deaths**	**RR (95% CI)**	**No. participants**	**No. deaths**	**RR (95% CI)**	**No. participants**	**No. deaths**	**RR (95% CI)**
Liver									
Never	26 544	132	0.81 (0.66, 0.99)	16 929	19	1.06 (0.63, 1.80)	9615	113	0.77 (0.62, 0.95)
Former	17 629	178	1.28 (1.08, 1.53)	10 028	29	1.87 (1.21, 2.90)	7601	149	1.19 (0.98, 1.44)
Occasional	73 104	497	1.00	51 413	68	1.00	21 691	429	1.00
Regular (<140 g/week)	15 865	161	1.31 (1.09, 1.58)	11 667	44	1.96 (1.32, 2.92)	4198	117	1.18 (0.95, 1.46)
Regular (≥140 g/week)	5271	193	4.03 (3.36, 4.83)	3708	75	8.07 (5.63, 11.56)	1563	118	3.10 (2.49, 3.87)
Respiratory									
Never	26 544	197	1.15 (0.97, 1.37)	16 929	26	0.95 (0.60, 1.51)	9615	171	1.19 (0.99, 1.43)
Former	17 629	175	1.22 (1.02, 1.45)	10 028	20	1.06 (0.64, 1.73)	7601	155	1.25 (1.03, 1.51)
Occasional	73 104	494	1.00	51 413	81	1.00	21 691	413	1.00
Regular (<140 g/week)	15 865	108	0.93 (0.75, 1.16)	11 667	18	0.95 (0.56, 1.62)	4198	90	0.93 (0.73, 1.18)
Regular (≥140 g/week)	5271	44	0.98 (0.71, 1.35)	3708	15	1.97 (1.10, 3.55)	1563	29	0.77 (0.53, 1.14)
External									
Never	26 544	59	1.30 (0.95, 1.77)	16 929	14	1.08 (0.58, 1.99)	9615	45	1.38 (0.97, 1.99)
Former	17 629	54	1.09 (0.80, 1.49)	10 028	14	1.15 (0.63, 2.08)	7601	40	1.06 (0.74, 1.53)
Occasional	73 104	170	1.00	51 413	53	1.00	21 691	117	1.00
Regular (<140 g/week)	15 865	76	1.39 (1.05, 1.84)	11 667	32	1.75 (1.10, 2.78)	4198	44	1.21 (0.84, 1.73)
Regular (≥140 g/week)	5271	43	1.86 (1.32, 2.64)	3708	18	2.31 (1.31, 4.06)	1563	25	1.63 (1.04, 2.55)
Other *									
Never	26 544	89	1.13 (0.87, 1.46)	16 929	17	1.24 (0.69, 2.24)	9615	72	1.11 (0.84, 1.48)
Former	17 629	72	1.10 (0.84, 1.45)	10 028	11	1.19 (0.61, 2.32)	7601	61	1.09 (0.81, 1.47)
Occasional	73 104	223	1.00	51 413	42	1.00	21 691	181	1.00
Regular (<140 g/week)	15 865	65	1.21 (0.91, 1.62)	11 667	15	1.39 (0.75, 2.59)	4198	50	1.18 (0.85, 1.63)
Regular (≥140 g/week)	5271	37	1.82 (1.26, 2.63)	3708	9	2.41 (1.11, 5.22)	1563	28	1.70 (1.12, 2.58)

Analyses are stratified by age-at-risk and district and adjusted for sex, education, smoking status, leisure-time physical activity and self-reported diabetes at baseline. Occasional drinkers are those who reported drinking alcohol on a less than monthly basis. Regular drinkers are those who reported drinking alcohol at least on a monthly basis. Those reporting to drink at least monthly but less than weekly are included in the <140g/week category. * Other alcohol-related causes include alcohol-related cancers (hepatobiliary and upper aerodigestive cancer), non myocardial infarction acute ischaemic heart disease, and ill-defined causes.

Webtable 13. Alcohol consumption versus non-alcohol-related mortality at ages 35-74 years, overall and by age-at-risk

	35-74 years			35-54 years			55-74 years		
**Cause of death, alcohol category**	**No. participants**	**No. deaths**	**RR (95% CI)**	**No. participants**	**No. deaths**	**RR (95% CI)**	**No. participants**	**No. deaths**	**RR (95% CI)**
All non-alcohol-related causes									
Never	26 544	2120	1.18 (1.12, 1.24)	16 929	355	1.50 (1.31, 1.71)	9615	1765	1.13 (1.06, 1.19)
Former	17 629	1894	1.20 (1.14, 1.27)	10 028	250	1.53 (1.32, 1.77)	7601	1644	1.16 (1.10, 1.23)
Occasional	73 104	5272	1.00	51 413	721	1.00	21 691	4551	1.00
Regular (<140 g/week)	15 865	1071	0.89 (0.83, 0.95)	11 667	180	1.09 (0.92, 1.30)	4198	891	0.86 (0.80, 0.92)
Regular (≥140 g/week)	5271	465	1.00 (0.91, 1.10)	3708	77	1.21 (0.95, 1.54)	1563	388	0.97 (0.87, 1.07)
Vascular									
Never	26 544	699	1.29 (1.17, 1.41)	16 929	105	2.13 (1.65, 2.76)	9615	594	1.20 (1.09, 1.32)
Former	17 629	674	1.32 (1.20, 1.44)	10 028	72	1.83 (1.39, 2.42)	7601	602	1.26 (1.15, 1.39)
Occasional	73 104	1648	1.00	51 413	172	1.00	21 691	1476	1.00
Regular (<140 g/week)	15 865	357	0.89 (0.79, 1.00)	11 667	73	1.54 (1.16, 2.05)	4198	284	0.81 (0.71, 0.92)
Regular (≥140 g/week)	5271	147	0.92 (0.78, 1.10)	3708	22	1.19 (0.75, 1.87)	1563	125	0.89 (0.74, 1.07)
Cancer									
Never	26 544	373	1.03 (0.91, 1.17)	16 929	87	1.23 (0.94, 1.60)	9615	286	0.98 (0.86, 1.13)
Former	17 629	275	1.10 (0.96, 1.26)	10 028	45	1.35 (0.97, 1.88)	7601	230	1.06 (0.91, 1.22)
Occasional	73 104	1029	1.00	51 413	185	1.00	21 691	844	1.00
Regular (<140 g/week)	15 865	191	0.87 (0.74, 1.02)	11 667	31	1.00 (0.67, 1.48)	4198	160	0.85 (0.71, 1.01)
Regular (≥140 g/week)	5271	79	1.06 (0.83, 1.34)	3708	12	1.36 (0.74, 2.52)	1563	67	1.01 (0.78, 1.31)
Renal									
Never	26 544	442	1.17 (1.04, 1.31)	16 929	71	1.34 (1.00, 1.80)	9615	371	1.14 (1.00, 1.29)
Former	17 629	420	1.21 (1.08, 1.36)	10 028	67	1.49 (1.12, 1.99)	7601	353	1.17 (1.03, 1.33)
Occasional	73 104	1026	1.00	51 413	168	1.00	21 691	858	1.00
Regular (<140 g/week)	15 865	177	0.85 (0.72, 1.00)	11 667	28	0.74 (0.49, 1.12)	4198	149	0.87 (0.73, 1.05)
Regular (≥140 g/week)	5271	91	1.08 (0.87, 1.35)	3708	15	0.87 (0.51, 1.50)	1563	76	1.12 (0.88, 1.43)
Respiratory									
Never	26 544	217	1.13 (0.96, 1.33)	16 929	18	1.68 (0.92, 3.08)	9615	199	1.10 (0.93, 1.30)
Former	17 629	173	0.99 (0.84, 1.18)	10 028	8	1.13 (0.52, 2.47)	7601	165	0.99 (0.83, 1.17)
Occasional	73 104	643	1.00	51 413	34	1.00	21 691	609	1.00
Regular (<140 g/week)	15 865	138	0.80 (0.67, 0.97)	11 667	5	0.55 (0.21, 1.44)	4198	133	0.82 (0.67, 0.99)
Regular (≥140 g/week)	5271	57	0.87 (0.66, 1.15)	3708	6	1.78 (0.72, 4.41)	1563	51	0.82 (0.61, 1.10)
Other medical									
Never	26 544	389	1.21 (1.07, 1.36)	16 929	74	1.38 (1.03, 1.84)	9615	315	1.17 (1.02, 1.34)
Former	17 629	352	1.23 (1.08, 1.39)	10 028	58	1.52 (1.12, 2.06)	7601	294	1.18 (1.03, 1.36)
Occasional	73 104	926	1.00	51 413	162	1.00	21 691	764	1.00
Regular (<140 g/week)	15 865	208	1.03 (0.88, 1.20)	11 667	43	1.17 (0.82, 1.66)	4198	165	1.00 (0.84, 1.19)
Regular (≥140 g/week)	5271	91	1.14 (0.91, 1.42)	3708	22	1.48 (0.93, 2.35)	1563	69	1.06 (0.82, 1.36)

Analyses are stratified by age-at-risk and district and adjusted for sex, education, smoking status, leisure-time physical activity and self-reported diabetes at baseline. Occasional drinkers are those who reported drinking alcohol on a less than monthly basis. Regular drinkers are those who reported drinking alcohol at least on a monthly basis. Those reporting to drink at least monthly but less than weekly are included in the <140g/week category.
